# Semiartificial Photoelectrochemistry for CO_2_-Mediated Enantioselective Organic Synthesis

**DOI:** 10.1021/jacs.5c02250

**Published:** 2025-04-15

**Authors:** Tessel Bouwens, Samuel J. Cobb, Celine W. S. Yeung, Yongpeng Liu, Guilherme Martins, Inês A. C. Pereira, Erwin Reisner

**Affiliations:** † Yusuf Hamied Department of Chemistry, University of Cambridge, Cambridge CB2 1EW, U.K.; ‡ Instituto de Tecnologia Química e Biológica António Xavier (ITQB NOVA), Universidade NOVA de Lisboa, Av. da República, 2780-157 Oeiras, Portugal

## Abstract

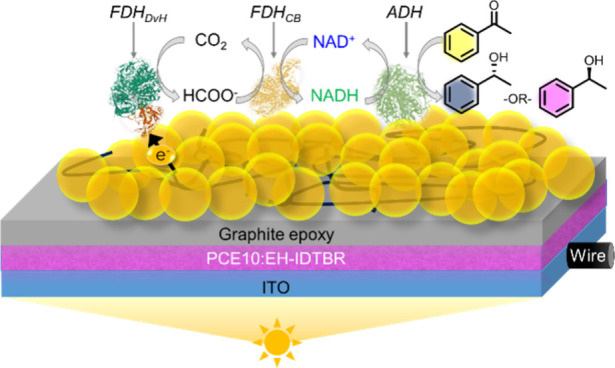

Photoelectrochemical (PEC) cells are under intensive development for the synthesis of solar fuels, but CO_2_ reduction typically only results in simple building blocks such as HCOO^–^. Here, we demonstrate that CO_2_-converting PEC cells can drive integrated enzymatic domino catalysis to produce chiral organic molecules by using CO_2_/HCOO^–^ as a sustainable redox couple. First, we establish a semiartificial electrode consisting of three enzymes co-immobilized on a high surface area electrode based on carbon felt covered by a mesoporous indium tin oxide (ITO) coating. When applying a mild cathodic potential (−0.25 V vs the reversible hydrogen electrode (RHE)), CO_2_ is reduced to HCOO^–^ using a W-formate dehydrogenase (FDH_NvH_) from *Nitratidesulfovibrio vulgaris* Hildenborough, which then enables the reduction of NAD^+^ to NADH by an NAD^+^-cofactor-dependent formate dehydrogenase from *Candida boidinii* (FDH_CB_). Subsequently, an alcohol dehydrogenase (ADH) uses NADH generated from CO_2_/HCOO^–^ cycling to reduce acetophenone to chiral 1-phenylethanol in good enantiomeric excess (93%) and conversion yields (38%). Depending on the specific ADH (ADH_S_ or ADH_R_), either (*S*)- or (*R*)-1-phenylethanol can be synthesized at pH 6 and 20 °C. To illustrate solar energy utilization, we integrate the three nanoconfined enzymes with a PEC platform based on an integrated organic semiconductor photocathode to allow for enantioselective synthesis (at +0.8 V vs RHE) based on a solar fuel device. This proof-of-principle demonstration shows that concepts and devices from artificial photosynthesis can be readily translated to precise and sustainable biocatalysis, including the production of chiral organic molecules using light.

Photosynthesis has long inspired scientists as it harnesses solar energy to convert simple building blocks (CO_2_, H_2_O) into complex organic chemicals (glucose) while releasing O_2_.^1,2^ Accordingly, artificial photosynthetic devices such as photoelectrochemical (PEC) cells aim to mimic this process, but they currently only produce relatively simple chemical fuels (e.g., H_2_, HCOO^–^, CO, CH_4_, ethylene, ethanol, or propanol).^3−7^ Current efforts to mimic the natural process have mainly focused on the light-dependent reactions in the thylakoid membrane, while the compartmentalized multienzyme machinery responsible for glucose synthesis from CO_2_ occurring in the light-independent stromal reactions has received limited attention.^8^ As mature PEC devices with high efficiency and stability are emerging, a growing opportunity arises to expand the reactivity space from simple fuels to more complex organic molecules by integrating catalytic cascade reactions in devices, thereby bringing us a step closer to truly mimicking the reactivity of natural photosynthesis.

State-of-the-art PEC cells provide an excellent platform to expand from classical solar fuels reactions (water splitting, CO_2_ reduction) to enantioselective organic synthesis, in line with our ongoing mission to integrate the fields of artificial photosynthesis and organic chemistry.^9^ While the recent renaissance of organic photoredox catalysis has made substantial progress in developing visible light responsive photocatalysts for many noteworthy reactions, it has not yet taken full advantage of the opportunities provided by chiral biocatalytic synthesis under benign, aqueous conditions and solid-state devices employing state-of-the-art semiconductors.

In this study, we aim to close this gap and develop a platform that uses solar energy to drive biocatalysis integrated into a PEC device for chiral organic synthesis. We employ evolutionarily optimized enzymes as biocatalysts to tackle the bottleneck of synthetic catalysts to overcome efficiency, selectivity, and reactivity issues.^10−12^ While semiartificial PEC cells employing enzymes are known, only the production of simple fuels such as H_2_,^13,14^ CO,^15^ HCOO^–^,^16,17^ and MeOH^18,19^ has been demonstrated. However, oxidoreductases provide an opportunity to catalyze an exciting diversity of reactions, including control of chemo-, regio-, and stereoselectivities, which outcompetes the possibilities provided by synthetic catalysts.^20−22^ To provide a glimpse of possibilities for the future, we demonstrate here that PEC cells producing classical solar fuels, such as HCOO^–^ from CO_2_, can be readily modified using cofactor-dependent enzymes for chiral synthesis using light (Figure 1).

**Figure 1:**
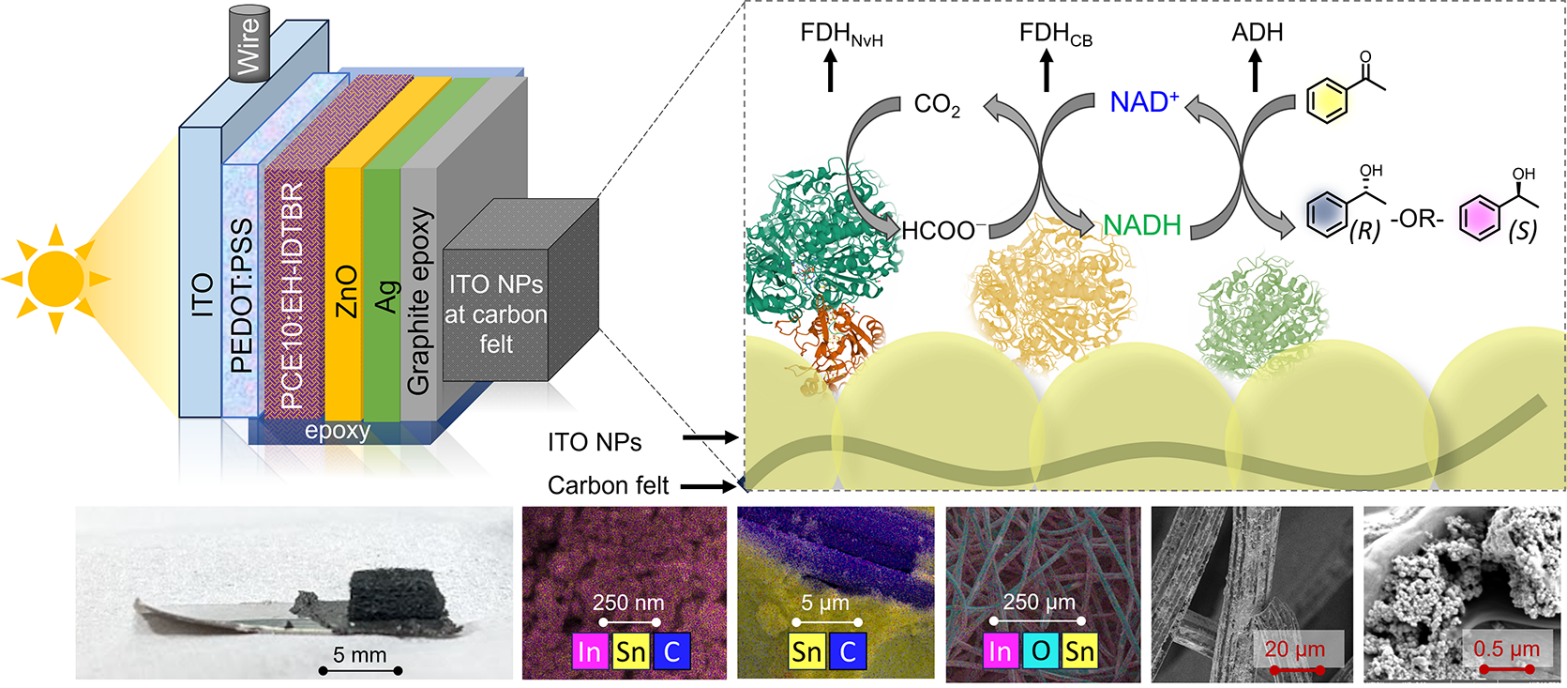
Schematic representation of the PEC-cascade system using CO_2_/HCOO^–^ as a mediator to generate chiral building blocks featuring FDH_NvH_, FDH_CB_, and ADH. All three enzymes are integrated into a Ti|ITO-CF scaffold (bottom left) for electrochemical characterization and an OPV-based photocathode, giving OPV|ITO-CF|FDH_NvH_/FDH_CB_/ADH. The SEM and EDX images of Ti|ITO-CF electrodes show the ITO NPs on CF.

The integrated enzyme cascade consists of three enzymes. The first enzyme is formate dehydrogenase (FDH_NvH_) from *Nitratidesulfovibrio vulgaris* Hildenborough (NvH),^23,24^ which is known to attach in an electroactive configuration to porous ITO electrodes via noncovalent interactions, allowing for CO_2_ reduction to HCOO^–^ with marginal overpotential and a *K*_M_ for CO_2_ of 0.42 mM.^25−27^ The next enzyme is the NAD^+^-cofactor-dependent formate dehydrogenase from *Candida boidinii* (FDH_CB_), which uses HCOO^–^ to catalyze the reduction of NAD^+^ to NADH. The last enzyme is an alcohol dehydrogenase (ADH), which can use the NADH produced from CO_2_/HCOO^–^ cycling to reduce pro-chiral acetophenone (selected as model building block for pharmaceutical applications^28,29^).

Porous metal oxide electrodes made from indium tin oxide (ITO) provide conductivity, a robust interaction with electroactive enzymes, and their porosity gives access to a high surface area to support large quantities of enzymes.^30−32^ However, the brittleness and conductivity constraints of porous ITO films only allow for film thicknesses up to approximately 50 μm.^33,34^ Therefore, we developed a conducting scaffold for enzyme immobilization in this study using commercially available carbon felt (CF) cuboids (5 × 5 × 3.2 mm^3^) coated with ITO nanoparticles. These macroscopic cuboids provide millimeter thickness and can be readily connected to a titanium foil (20 × 10 mm^2^) using graphite epoxy (Figures S1–S4, further discussion in the Supporting Information). These Ti|ITO-CF electrodes can be used as a (dark) cathode or integrated into a photocathode (see below). The maximum loading of ITO NPs onto CF was determined (Figure S1), and the CF cuboids demonstrated an apparent porosity of 78%, close to the reported porosity of these CF materials (>80%). Ti|ITO-CF was characterized by SEM and EDX (Figure 1, Figures S2–S4), which show the individual ITO NPs and the presence of In, Sn, and O on the carbon fibers.

FDH_NvH_ (125 pmol) was then dropcast onto the Ti|ITO-CF electrode (0.25 cm^2^) and analyzed using protein film voltammetry (PFV) in an aqueous electrolyte solution containing MOPS (13.5 mL, 0.1 M, pH 6), DMSO (1.5 mL), NAD^+^ (1 mM), and acetophenone (50 mM), saturated with CO_2_ at ambient temperature (20 °C, Table S1). The PFV response shows an onset of catalytic current for CO_2_ reduction close to 0 V vs the reversible hydrogen electrode (RHE), as expected for the reversible CO_2_ to HCOO^–^ biocatalyst (Figure 2).^32^ During the back scan, an anodic wave is observed with a peak current at approximately +0.2 V vs RHE, which is only present upon scan reversal following the catalytic cathodic wave (Figure 2b). This observation suggests that this cathodically induced anodic wave is due to the oxidation of HCOO^–^ produced from the reduction of CO_2_ in the porous stationary Ti|ITO-CF|FDH_NvH_ electrode. In the absence of CO_2_, no catalytic currents are observed (Figure 2a).

**Figure 2:**
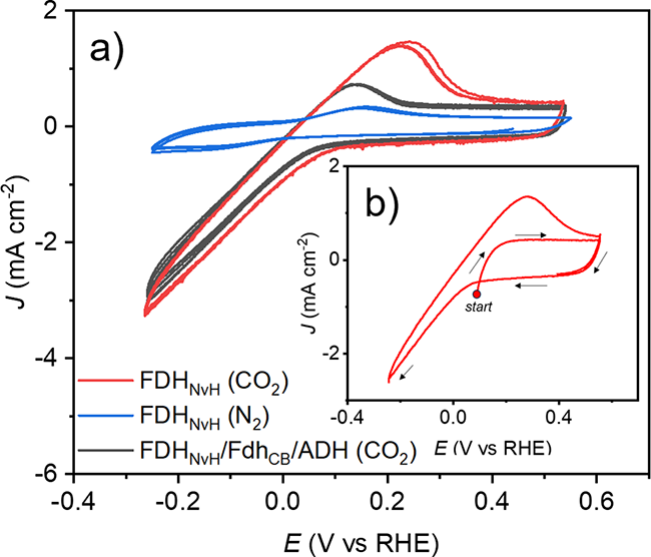
(a) Protein film voltammetry (PFV) scans of FDH_NvH_ (red and blue traces) and the 3-enzyme cascade (black trace) under CO_2_ (red and black traces) or N_2_ (blue trace). (b) PFV analysis of FDH_NvH_, starting at +50 mV to 550 mV to −250 mV and back (red trace). PFV scans recorded using 10 mV s^–1^ in electrolyte comprised of MOPS (13.5 mL, 0.1 M, pH 6), NAD^+^ (1 mM), acetophenone (50 mM), and DMSO-*d*_6_ (1.5 mL) (Table S1).

Next, we studied the electrochemical response with all three enzymes, using FDH_NvH_ (125 pmol), FDH_CB_ (1 nmol), and ADH (5 nmol) dropcast onto Ti|ITO-CF (0.25 cm^2^). Note that solely FDH_NvH_ is an electroactive enzyme receiving electrons directly from Ti|ITO-CF, whereas FDH_CB_ and ADH rely on HCOO^–^ and NADH as an electron source. In a CO_2_-saturated solution with NAD^+^ and acetophenone we observe a qualitatively similar voltametric cathodic response for Ti|ITO-CF|FDH_NvH_/FDH_CB_/ADH compared to Ti|ITO-CF|FDH_NvH_, but the current density of the anodic wave at +0.2 V vs RHE is significantly reduced (Figure 2a). This observation supports the idea that HCOO^–^ is readily consumed by FDH_CB_ and used to generate the NADH cofactor that is needed to produce the chiral alcohol product.

Protein film chronoamperometry (PF-CA) was performed with ITO-CF|FDH_NvH_ at an applied potential of −0.25 V vs RHE (Figure S5), showing quantitative conversion of CO_2_ into HCOO^–^ (Faradaic efficiency, FE_HCOO^–^_ = 99%; determined by ^1^H NMR spectroscopy, Figures S6–S8). PF-CA with all three enzymes produces 1-phenylethanol (PE), demonstrating that the cascade is functional electrochemically (FE_HCOO^–^_ = 72% and FE_PE_ = 27%, FE_total_ = 99%).

The turnover number of ADH (TON_ADH_) for 1-phenylethanol synthesis is estimated from PF-CA at approximately 5 × 10^3^ after 12 h (Figures S5, Figures S9 and S10). The cascade generates chiral 1-phenylethanol in high conversions after 12 h (38 ± 8%, Table S2, Figure S7f). Depending on the choice of ADH (Figures S11 and S12), we were able to produce both the (*S*)-enantiomer (enantiomeric excess, *ee* = 93%, determined by chiral HPLC) or the (*R*)-enantiomer (*ee* = 59%) (Figure 2b, Figures S13 and S14).

Isotopic labeling experiments using ^13^CO_2_ with the Ti|ITO-CF|FDH_NvH_/FDH_CB_/ADH electrode at an applied potential of −0.25 V vs RHE for 4 h confirm the origin of HCOO^–^ solely from CO_2_ (^1^H NMR, δ = 8.44 ppm, doublet, *J*_C–H_ = 194 Hz, Figure S15). A control experiment using ITO-CF|FDH_NvH_/FDH_CB_/ADH without NAD^+^ resulted in negligible amounts of 1-phenylethanol (Figure S16).

The FE and *ee* for Ti|ITO-CF|FDH_NvH_/FDH_CB_/ADH are comparable to previous work employing electrocatalytic enzymatic cascades based on NAD(P)H-recycling using ferrodoxin NADP^+^ reductase.^22,35,36^ Synthetic cofactor regeneration strategies have also been applied previously, e.g., using Cp*Rh(bpy)-derived mediators, but these systems suffer from side reactions, such as H_2_ formation (FE_NADH_ < 86%).^18,37−45^ Our semiartificial platform operates selectively (quantitative FE_NADH_, no side-products detected) with a high *k*_cat_(NAD^+^)_FDH_ = 8400 h^–1^, whereas Cp*Rh(bpy)-derived mediators only reach *k*_cat_(NAD^+^)_Rh_ = 36 h^–1^.^46^ Electrocatalytic production was also integrated with ADH-promoted synthesis of 1-propanol from 1-propanal via Cp*Rh(bpy)-mediated cofactor regeneration,^44^ but this system requires a flow-through electrolytic cell with a negative potential of −1.6 V vs RHE.

The interfacial electron transfer kinetics were investigated by electrochemical impedance spectroscopy (EIS)^47^ over the same potential range used for the PFV scans with 0.1 V intervals and a sinusoidal perturbation of 15 mV. Quantitative analysis with Nyquist plot fitting (Figures S17–S20) has previously been validated for electroenzymatic reactions involving H_2_ase and FDH_NvH_.^48−50^ The values for the resistance *R*_*e*_ remain similar across different systems; thus the values of time constant *τ*_*e*_, defined by *τ*_*e*_ = *R*_*e*_ × *C*_*e*_, are driven by the distinct differences in *C*_*e*_ values. The time constant describes all of the processes occurring within the measurement time domain. The addition of FDH_CB_ and FDH_CB_/ADH leads to a smaller *τ*_*e*_, suggesting that the CO_2_/HCOO^–^-cycling for Ti|ITO-CF|FDH_NvH_/FDH_CB_/ADH is faster than the single process at the Ti|ITO-CF|FDH_NvH_ electrode (further discussion in the Supporting Information).^51^

After establishing the semiartificial electrocatalytic domino system, we integrated an organic photovoltaic (OPV)-based PEC system based on a recently established π-conjugated organic semiconductor PEC platform.^50^ Specifically, we employed an OPV device based on PCE10:EH-IDTBR that is encapsulated by graphite epoxy to (i) protect the OPV from the aqueous electrolyte solution and (ii) integrate the ITO-CF electrode scaffold (Figure 1).^52^ The ITO-CF cuboid is connected to the OPV using graphite-epoxy paste, and the photocathode is wired to a metal rod. The enzymes were drop-cast onto the OPV|ITO-CF using the same procedure as that for the electrochemical studies on Ti|ITO-CF.

The OPV|ITO-CF|FDH_NvH_/FDH_CB_/ADH photocathode displays an onset potential of 1 V vs RHE with a high photocurrent density of approximately −6 mA cm^–2^ at 0 V vs RHE under standard solar spectrum air mass 1.5 global (AM1.5G) irradiation at 20 °C (pH 6, Figure 3a, Figure S21). PF-CA showed a relatively stable current density of ∼1 mA cm^–2^ at +0.8 V vs RHE during 12 h AM1.5G irradiation (Figure 3b). The generation of the intermediate HCOO^–^ and (*S*)-1-phenylethanol was followed by ^1^H NMR spectroscopy (Figure 3c, Figures S22 and S23). After 12 h, a FE_PE_ of 10% with a FE_HCOO^–^_ of 54% was obtained. Our OPV|ITO-CF|FDH_NvH_/FDH_CB_/ADH photocathode therefore demonstrates solar-powered recycling of NADH using CO_2_/HCOO^–^ as a mediator for the enantioselective synthesis of (*S*)-1-phenylethanol (TON_ADH_ of approximately 1.2 × 10^3^, Figures S23 and S24). The longevity of the current domino PEC system is likely limited by NADH degradation during irradiation (Figure S25).

**Figure 3:**
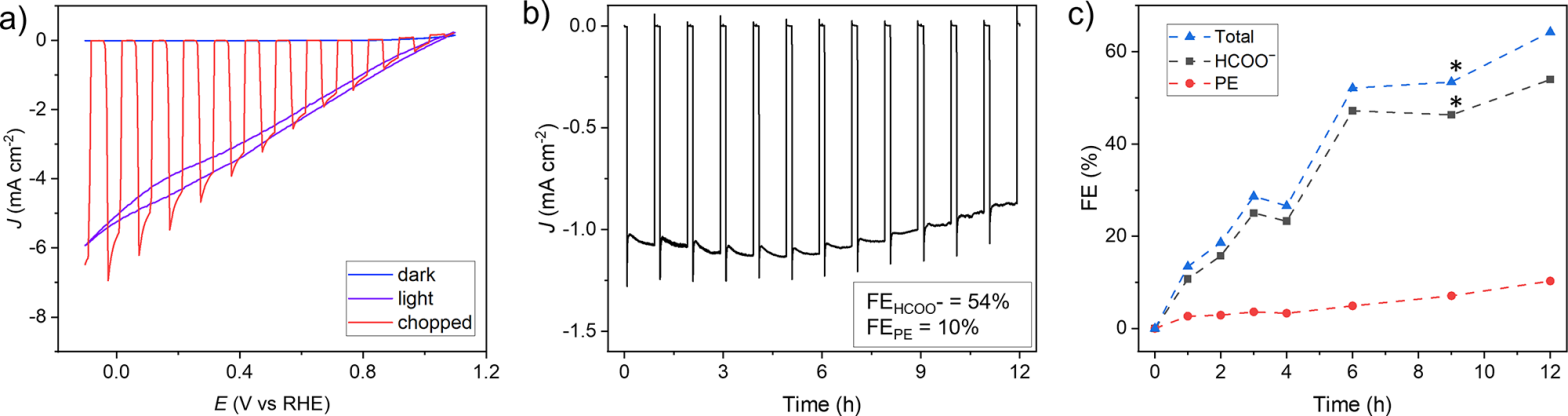
Production of chiral (*S*)-1-phenylethanol using a PEC system: OPV|ITO-CF|FDH_NvH_/FDH_CB_/ADH. (a) PFV scans in dark, light, and chopped with on/off cycles of 10 s. (b) Chopped light PF-CA at +0.8 V vs RHE with 50 min on- and 10 min off-cycles. NMR samples were collected after *t* = 0, 1, 2, 3, 4, 6, 9, and 12 h. (c) FE_PE_, FE_HCOO^–^_, and FE_Total_. *Calculated from deconvoluted NMR signals.

In summary, we have demonstrated the integration of three enzymes into a state-of-the-art PEC cell using CO_2_ as a redox mediator to produce NADH to drive the synthesis of chiral organics (both (*R*)- and (*S*)-enantiomers). This strategy provides a possible platform to leverage enzyme-driven photoelectrochemistry to synthesize chiral organic chemicals from simple substrates for the chemical and pharmaceutical industry in the future.^28,29,53^ This principle can be readily adopted by contemporary solar fuel devices producing (i) other fuels, e.g., H_2_ using a cofactor-dependent hydrogenase,^13,54^ and (ii) other photocathode materials, e.g., encapsulated silicon, copper oxide, and lead halide perovskite.^4,5,55^ This work also intends to inspire and motivate efforts to connect the materials- and device-focused artificial photosynthesis community with organic chemists employing biocatalysis and photoredox catalysis.

## Data Availability

Experimental data of this study can be accessed through the University of Cambridge data repository: 10.17863/CAM.117123.

## Supporting Information Available

Experimental procedures, material characterization, and additional spectroscopic data (PDF)
