# Supplementary material for: Semiartificial Photoelectrochemistry for CO2-Mediated Enantioselective Organic Synthesis
Source: J Am Chem Soc. 2025 Apr 15;147(16):13114–9. doi: 10.1021/jacs.5c02250 (PMC12022976; doi:10.1021/jacs.5c02250)
Supplement: Supplementary file 1 — ja5c02250_si_001.pdf [file ja5c02250_si_001.pdf]

Supporting Information

Semi-artificial photoelectrochemistry for CO_2-mediated enantioselective organic synthesis

Tessel Bouwens[1], Samuel J. Cobb[1], Celine W. S. Yeung[1], Yongpeng Liu[1], Guilherme Martins[2], Inês A. C. Pereira[2], Erwin Reisner[1]*

[1] Yusuf Hamied Department of Chemistry, University of Cambridge, Cambridge CB2 1EW, U.K.

[2] Instituto de Tecnologia Química e Biológica António Xavier (ITQB NOVA), Universidade NOVA de Lisboa, Av. da República, 2780-157 Oeiras, Portugal.

*Email: reisner@ch.cam.ac.uk

Experimental procedures

Materials and Methods. All chemicals were used without purification, unless stated otherwise. The chemicals β-nicotinamide adenine dinucleotide hydrate (NAD^+, purity ≥ 97%), β-nicotinamide adenine dinucleotide reduced disodium salt hydrate (NADH, purity ≥ 98%), sodium formate (HCOONa, 99.5%), 1,4-dithiothreitol (DTT, BioXtra >99.5%), 3-(*N*-morpholino)propanesulfonic acid (MOPS-H, Bioxtra >99.5%, 3-(*N*-morpholino)propanesulfonic acid sodium salt (MOPS-Na, BioXtra >99.5%) were purchased from Sigma Aldrich (Merck). The water used to make electrolytes and other solutions was deionized/demineralized by a Simplicity UV MilliQ system (18 MΩ cm). The gases N_2 and CO_2 (2% Methane/Carbon dioxide) were received from BOC Limited. FDH_{NvH} was expressed and purified from *Nitratidesulfovibrio vulgaris* Hildenborough (NvH) according to a previous report.[1] Note that *Nitratidesulfovibrio vulgaris* Hildenborough was formerly called *Desulfovibrio vulgaris* Hildenborough. ADH was obtained from Johnson Matthey as a gift and FDH_{CB} purchased from Sigma Aldrich (Merck; Roche Diagnostics GmbH, 80 U, 0,54 U/mg L). The membrane filters to purify FDH_{CB} were obtained from Sigma Aldrich (Merck) (Amicon Ultra 0.5 mL, Ultracel 30K Membrane). The products were analyzed using ^1H NMR spectroscopy (400 MHz, Bruker, Neo Prodigy Spectrometer) and HPLC (Waters 1525 equipped with a Waters 2489 UV/Visible Detector 254 nm using a Daicel CHIRALPAK® IB N-5, 4.6 x 250 mm 5 µm, applying water/acetonitrile = 80:20 as mobile phase).

Preparation of ITO NPs on carbon felt (ITO-CF). The ITO NPs (50 nm, 100 mg) (prepared according to previous work[2]) were suspended in EtOH (715 µL) and glacial acetic acid (285 µL) by cycles of sonication (30 min, at 70% sonication strength) and vortex mixing (1 min). The temperature of the sonication bath was kept at <35 °C. These cycles were continued until the particles were completely suspended, which typically required 4–6 cycles. The CF was cut into 10 cuboids (0.5 × 0.5 cm^2) and cleaned by sonication in EtOH/water = 1:4. The CF cuboids were dried in an oven (130 °C, 2 hours). The ITO NP suspension was dropcast onto the CF cuboids (~100 µL, until the cuboid started leaking). The ITO NP on CF was annealed at 400 °C for 30 min with a ramp rate of 4 °C min^{-1} from room temperature. The obtained ITO-CF cuboids changed color from blue to slight yellow/green. The ITO-CF cuboids were used without any further treatment.

Preparation of the Ti|ITO-CF electrodes. The Ti|ITO-CF cuboids described above were applied to connect to cuboids to titanium foil (Ti, 20 × 10 mm^2) using a graphite epoxy paste. The paste was prepared by thoroughly mixing Araldite Standard 2-part epoxy and graphite powder in a 4:3 mass ratio.[3,4] A layer of the epoxy graphite paste was applied upon the Ti foil covering 10 × 10 mm^2 of the surface. The Ti|ITO-CF cuboids was pressed carefully into the paste and the sample was left to dry for 24 hours before use.

Assembly of ITO-CF|FDH_{NvH} electrode. To activate the FDH_{NvH} enzyme, a solution of dithiothreitol (DTT, 80 mM) was prepared in Tris-HCl (20 mM, pH 7.5) inside a N_2 filled glovebox. FDH_{NvH} (2.5 µL, 125 pmol, 50 mM) was suspended in the DTT solution (10 µL, 800 pmol) followed by 20 min activation. The FDH_{NvH} solution containing the activated enzyme was dropcast onto the ITO-CF electrode, followed by incubation for 2–3 min.

Assembly of ITO-CF|FDH_{NvH}/FDH_{CB} electrode. First, to activate FDH_{NvH} a solution of dithiothreitol (DTT) (80 mM) was prepared in Tris-HCl (20 mM, pH 7.5). The FDH_{NvH} (2.5 µL, 125 pmol, 50 mM) was suspended in the DTT solution (10 µL, 800 pmol) followed by 20 min activation inside a N_2 filled glovebox. The FDH_{NvH} solution containing the activated enzyme was dropcast onto the ITO-CF electrode, followed by incubation for 2–3 min. Then, a solution of FDH_{CB} (60 mM) was prepared in Tris-HCl (20 mM, pH 7.5), and filtered using centrifugation

at 11,000 rpm, 3×10 min at 4 °C. The FDH_{CB} (16.5 µL; 60 mM; 1.0 µmol) was dropcast onto the same ITO-CF electrode as the FDH_{NvH} (after 2–3 min), followed by incubation for 2–3 min. The two enzymes were dropcast in the same consecutive order (first: FDH_{NvH} second: FDH_{CB}) onto the ITO-CF electrode 2–3 min apart.

Assembly of ITO-CF|FDH_{NvH}/FDH_{CB}/ADH electrode. First**,** to activate FDH_{NvH} a solution of dithiothreitol (DTT) (80 mM) was prepared in Tris-HCl (20 mM, pH 7.5). The FDH_{NvH} (2.5 µL, 125 pmol, 50 mM) was suspended in the DTT solution (10 µL, 800 pmol) followed by 20 min activation inside a N_2 filled glovebox. The FDH_{NvH} solution containing the activated enzyme was dropcast onto the ITO-CF electrode, followed by incubation for 2–3 min. Then, a solution of FDH_{CB} (60 mM) was prepared in Tris-HCl (20 mM, pH 7.5), and filtered using centrifugation at 11,000 rpm, 3×10 min at 4 °C. The FDH_{CB} (16.5 µL; 60 mM; 1.0 µmol) was dropcast onto the same ITO-CF electrode as the FDH_{NvH} (after 2–3 min), followed by incubation for 2–3 min. Finally, the ADH enzyme solution (50 µL, 10 mg mL^{-1}, 500 µg) was dropcast onto the ITO-CF electrode, followed by incubation for 2–3 min.[5] The three enzymes were dropcast in the same consecutive order (first: FDH_{NvH} second: FDH_{CB} third: ADH) onto the ITO-CF electrode 2–3 min apart.

MOPS buffer. Both MOPS-H (2.57 g; 12.3 mmol) and MOPS-Na (1.79 g; 7.74 mmol) were dissolved in water (18 MΩ cm, 180 mL) and water was added until a volume of 200 mL was reached. The MOPS buffer (0.1 M) was then saturated with CO_2 and adjusted to pH = 6.

Electrolyte solution. Phenylacetone (0.09 mL, 0.75 mmol) was dissolved in DMSO-d^6 (1.41 mL, 1.55 g) and added to NAD^+ (15 µmol) dissolved in MOPS buffer (pH 7, 0.1 M, 13.50 mL) to give 15 mL of electrolyte solution used in electrocatalysis and PEC experiments. The electrolyte solution was saturated with CO_2 (unless stated otherwise) using a gentle stream of CO_2 gas for 30 min, giving a final pH 6. The electrolyte was prepared freshly for each experiment and the constituents are listed in Table S1.Only 0.6 mL of this 15 mL electrolyte solution was used when employing the small cell set-up (see below).

Electrochemical Experiments.

The electrochemical measurements were performed using a Biologic VSP-300, 4 Channel potentiostat electrochemical workstation in a two-compartment H-cell separated by a bipolar membrane. The bipolar membrane was employed to prevent crossover of phenylacetone from the catholyte to the anolyte. Ti|ITO-CF modified with enzymes was used as the working electrode, carbon felt (2 × 1 cm^2) connected to a metal rod was used as the counter electrode, and a Ag/AgCl reference electrode (in 3.5 M KCl, BASi MW-2030) was used. The potentials were converted to the RHE scale using Equation (1) at a temperature of 298 K:[6]

$$V_{RHE} = V_{Ag/AgCl} + 0.059 \times pH + 197\,mV\ (298\,K) \quad (1)$$

Two sizes of electrochemical cells were used. The large cell set-up (15 mL) was used in most experiments and consisted of a working compartment with 15 mL electrolyte solution and a counter compartment with 9 mL electrolyte solution (Figure 23a). The small cell set-up (0.6 mL) was used in PF-CA for precise determination of the enantiomeric excess (ee) and consisted of a working compartment with 0.6 mL electrolyte solution and a counter compartment with 2 mL electrolyte solution (Figure 6). A leakless miniature Ag/AgCl reference electrode (Length: 65 mm, diameter: 2 mm, ET072-1, eDAQ) was used in the small cell.

The faradaic efficiency (FE) was determined by multiplying the amount of product (either $HCOO^-$ or 1-phenylethanol, in mol) with $Z_{electrons}$ representing the number of electrons required

per molecule of product ($Z_{electrons}$ = 2) and Faraday constant 96,485 C mol^{-1}. This value is divided by the total amount of charge passed (Q), determined by integrating the current trace over a defined period (Equation 2).

$$FE\ (\%) = \frac{n_{product} \times Z_{electrons} \times F}{Q} \times 100 \quad (2)$$

The turnover number (TON) for 1-phenylethanol produced by ADH_R and ADH_S was calculated according to Equation 3 and the turnover frequency (TOF) according to Equation 4.

$$TON_{ADH} = \frac{moles\ of\ 1\text{-}phenylethanol}{moles\ of\ ADH} \quad (3)$$

$$TOF_{ADH}(h^{-1}) = TON_{ADH}/(time\ (h)) \quad (4)$$

The enantiomeric excess (*ee*) was determined using Equation 5, where F_R is the moll fraction of the (*R*)-enantiomer and (*S*)-enantiomer.

$$\%ee = |F_R - F_S| \times 100\% \quad (5)$$

Electrochemical Impedance Spectroscopy (EIS). EIS experiments were performed as described in previous work involving H_2ase and FDH_{NvH}.[7–9] EIS measurements were conducted in a two-chamber electrochemical cell with a 3-electrode configuration: an ITO-CF working electrode, a carbon felt counter electrode, and a Ag/AgCl reference electrode. The anaerobic electrolyte (15 mL) contains CO_2-saturated MOPS buffer (0.1 M, pH 6). Impedance response was recorded at −0.8, −0.7 −0.6, −0.5, −0.4, −0.3, −0.2, −0.1, 0.0 V vs Ag/AgCl on a BioLogic VSP potentiostat with frequency ranges from 100 kHz to 50 mHz and a 15 mV sinusoidal amplitude. Impedance data were fitted with equivalent circuits using modeling software ZView2 (Scribner Associates). The EIS data for ITO-CF was fitted using one equivalent circuit representing the double layer only (no catalysis). The EIS data recorded in the presence of enzymes were fitted with an additional circuit representing the electrocatalysis (Figure S17a).

Photoelectrochemical experiments. Conventional structure OPVs (PCE10:EH-IDTBR bulk heterojunction) were fabricated as previously reported.[6] EH-IDTBR (CAS: 2102510-60-9) is a non-fullerene acceptor, whereas PCE10 (CAS: 1469791-66-9) functions as donor. The ITO-CF cuboid (5 × 5 × 3.2 mm^3) is connected to the OPV using graphite-epoxy paste and the whole device is wired to a metal rod. The three enzymes were activated and dropcast as explained above. The OPV|ITO-CF|FDH_{NvH}/FDH_{CB}/ADH was submerged into 15.5 mL (to compensate for the t = 0 NMR sample) electrolyte comprised of MOPS buffer (14 mL, 0.1 M, pH 6), NAD^+ (15.5 µmol, 1 mM), acetophenone (0.77 mmol, 50 mM), d^6-DMSO (1.6 mL, 10% v/v). The counter compartment contains MOPS buffer (9 mL, 0.1 M, pH 6). Both working and counter compartment were saturated with CO_2 before the organic photocathodes were characterised using a Newport Oriel 67005 solar light simulator with an AM 1.5G optical filter (1 sun, 100 mW cm^{-2}). To calibrate the light intensity to AM 1.5G (100 mW cm^{-2}) a certified silicon reference solar cell RS-OD4 was applied. The *J*-V scans were conducted using a source meter (Keithley 2635) at a scan rate of 10 mV s^{-1} in 5 mV steps between −0.1 V and 1.1 V vs. RHE. We recorded PFV under continuous, chopped and no illumination (5 seconds

on/off cycles using a shutter), whereas the electrolyte was being stirred constantly. The chronoamperometry (PF-CA) experiments were performed at +0.8 V vs. RHE with 50 min on and 10 min off cycles. After 60 min, a sample from the electrolyte was taken (0.5 mL). Then, another PF-CA experiment was performed using the same procedure. The PF-CA and sampling were carried out at t = 0, 1, 2, 3, 4, 6, 9, 12 hours. The ^{1}H NMR samples were prepared by adding internal standard (100 µL) comprised of D_2O with trimethylsilylproanoic acid (TMSP) 0.75% (w/w) to the electrolyte sample (500 µL).

Additional Discussions

Discussion and characterization of the ITO-CF materials. Porous metals oxide electrodes have recently demonstrated their applicability for the direct electron transfer immobilization of enzymes,[2,10,11] offering a highly porous and conducting scaffold that can support large quantities of enzyme. However, these materials are limited to thickness dimensions on the scale of 10s to 100s of µm and their mechanical properties (namely brittleness) make further increase of these dimensions challenging. Carbon felt materials are ideally suited to increase the electrode's film thickness, but they do not possess pore sizes (pores>µm) or surface terminations (hydrophobic and generally negatively charged) that are compatible with enzymes. For this reason, ITO sintered onto carbon felt was developed in this work. The electrode was saturated with a dispersion of ITO NPs (40 nm, 100 mg mL^{-1} in 5:1 MeOH: AcOH), and the solution volume required plotted against electrode volume (Figure S1). This resulted in an apparent porosity of 78%, close to the reported porosity of these materials (>80%) and formed a calibration curve for the loading of differently sized electrodes. An ITO loading of ~6 mg on a standard 5×5×3.18 mm^3 electrode was used in this study. The electrodes containing ITO NPs dropcast on CF were allowed to dry and then sintered at 400 °C for 1 h (ramp rate 4 °C min^{-1}), after which carbon fibers that were mostly coated with a thin mesoporous ITO layer were observed by SEM (Figure S2).

Additional Discussion of EIS results. To gain deeper insights into the electron transfer mechanisms, electrochemical impedance spectroscopy (EIS) was performed over the same potential range used for the PFV scans with 0.1 V intervals and a sinusoidal perturbation of 15 mV. Quantitative analysis of the impedance data was conducted by fitting the Nyquist plot of impedance response (Figure S18) to equivalent circuit models (Figure S17a). The models incorporated key elements, including a series resistor (R_S), double-layer capacitor (C_{dl}), charge transfer resistor (R_{ct}), Warburg impedance (Z_W), electron transfer resistor (R_e), and electron transfer capacitance (C_e). This equivalent circuit framework has been previously validated for electroenzymatic reactions involving H_2ase and FDH_{NvH},[7,9,12] and is well-suited for the system investigated in this study.

The Randles circuit incorporating the Z_W (Figure S17a)[13] was used to model the impedance of electrodes without catalytic current, including both bare ITO NPs electrodes and enzyme hybrid electrodes prior to the onset potential. After the onset potential, enzyme hybrid electrodes were fitted using a two-parallel RC circuit model in series (Figure S17a).[14,15] The potential-independent R_{ct} suggests that the resistivity of the electrical double layer is primarily determined by the ITO NPs electrode due to the high porosity of mesoporous metal oxide electrodes.[16] Interestingly, the C_{dl} exhibits distinct behaviors across different experimental groups. The presence of FDH_{NvH} on the ITO NPs electrode does not affect the electrical double layer, with C_{dl} remaining potential-independent at around 100 µF for both the Ti|ITO-CF and Ti|ITO-CF|FDH_{NvH} electrodes. However, the addition of FDH_{CB} causes a significant change

below + 50 mV vs. RHE, leading to a two-order-of-magnitude increase in C_{dl}, reaching approximately 10 mF for Ti|ITO-CF|FDH_{NvH}/FDH_{CB} and Ti|ITO-CF|FDH_{NvH}/FDH_{CB}/ADH electrodes.

Regarding R_e, only the Ti|ITO-CF|FDH_{NvH} electrode displays potential dependence, with R_e increasing linearly with cathodic bias. In comparison to the two FDH-modified electrodes, the incorporation of ADH leads to an increase in R_e, likely due to the intrinsic resistive properties of the ADH. Moreover, both Ti|ITO-CF|FDH_{NvH}/FDH_{CB} and Ti|ITO-CF|FDH_{NvH}/FDH_{CB}/ADH electrodes exhibit lower C_e than for FDH_{NvH}, suggesting that surface charge accumulation is decreased by the consumption of produced formate. The RC time constant describing electron transfer (τ_e) was determined by multiplying R_e and C_e (Figure S17f).[17] The trend observed for τ_e mirrors that of C_e, as the values of R_e remain similar across the systems, while the variations in τ_e are driven by the distinct differences in C_e values. The addition of FDH_{CB} and FDH_{CB}/ADH lead to a smaller τ_e, suggesting that the CO_2/$HCOO^-$cycling for Ti|ITO-CF|FDH_{NvH}/FDH_{CB}/ADH is faster than the single process at the Ti|ITO-CF|FDH_{NvH} electrode.[18]

Table S1. The constituents of the electrolyte solution used in this work to make 15 mL electrolyte saturated with CO_2 (pH 6). DMSO is required to solubilize acetophenone in the aqueous electrolyte. *In case of PF-CA using the small cell (Figure S6), 0.6 mL of the 15 mL electrolyte mixture was used.

Chemicals	c (mol L^{-1})	Volume (mL)	m (g)	n (mol)	ρ (g cm^{-3})	M_W (g mol^{-1})
$NAD^+ \cdot H_2O$	1.00×10^{-3}		6.80×10^{-3}	1.50×10^{-5}		663
Acetophenone	5.00×10^{-2}	8.77×10^{-2}	9.03×10^{-2}	7.50×10^{-4}	1.03	120
DMSO-d^6	1.32	1.41	1.55	1.99×10^{-2}	1.10	78.1
MOPS buffer	1.00×10^{-1}	1.35×10^{1}				

Table S2. Summary of the PF-CA results with the error in brackets. N = 3–4. PE = 1-phenylethanol.

Entry	*V* (mL)	CA time (h)	Conversion PE (%)	FE_{HCOO^-} (%)
1	0.6	2	2.6 (±0.1)	3.2 (±9)
2	0.6	4	8.1 (±3.0)	0.1
3	0.6	8	14 (±3.8)	11
4	0.6	12	38 (±8.1)	18
5	15	12	3.7	72

Table S3. Comparison of electrochemical, photoelectrochemical, and photochemical biosynthetic systems.

• **Enzyme**<br>• **Support**<br>• **NAD(P)H recycling**	**Product**	**Performance**	**Ref**
Electrochemical			
FDH_{NvH}/FDH_{CB}/ADH<br>ITO-CF|Ti<br>Enzymatic: FDH_{CB}	1-Phenylethanol	TON = 5.0×10^3<br>TOF = 4.1×10^2 h^{-1}<br>FE_{Total} = 99%<br>Ee = 93%	This work
FNR/GlDH<br>ITO|Ti<br>Enzymatic: FNR	L-glutamate	TON = 4.1×10^4<br>TOF = 5.1×10^3 h^{-1}<br>Yield = 93.7%	19
FNR/GlDH<br>ITO NP<br>Enzymatic: FNR	L-glutamate	ITO-NP|ITO-glass<br>TON 1.8×10^3<br>Rate = 0.14 mmoles h^{-1} cm^{-2}<br><br>ITO-NP|Ti<br>TON = 5.0×10^3<br>Rate = 0.13 h^{-1} cm^{-2}	20
ADH<br>ZIF-8<br>Cp*Rh(bpy)-mediator	Fufuryl alcohol	Yield: 96.4% (24h)	21
ADH<br>N/A<br>Pd-reactor	n-Propanol	TON = 1.3×10^5<br>TOF = 5.1×10^4 h^{-1}	22
ADH<br>N/A<br>molybdenum sulfide (a-MoS_x)	Benzyl alcohol	TON = 4.4×10^2<br><br>TOF = 1.3×10^2 h^{-1}	23
ADH<br>CoOc polymer<br>CoOc polymer|Carbon paper	MeOH<br><br><br>Propanol	TON = 3.9×10^3<br>TOF = 1.6×10^2 h^{-1}<br><br>TON =3.1×10^3<br>TOF =131×10^2 h^{-1}	24
FNR/Ccr<br>C-electrode|V^{2+}-PVA V^{2+}- Enzymatic: FNR	ethylmalonyl-CoA	TON = 2.8×10^3<br>TOF = 4.0×10^2 h^{-1}<br>Rate = 0.14 µmol cm^{-2} .h^{-1}<br>FE: 83%	25
ADH<br>N/A<br>Cp*Rh(bpy)-mediator	*n*-propanol	TON 3.1×10^6<br>TOF 1.3×10^4 h^{-1}<br>Rate: 9.66 mM h^{-1}	26

• **Enzyme**<br>• **Support**<br>• **NAD(P)H recycling**	**Product**	**Performance**	**Ref**
Photoelectrochemical			
FDH_{NvH}/FDH_{CB}/ADH<br>OPV|ITO-CF<br>Enzymatic: FDH_{CB}	1-Phenylethanol	TON = 1.2× 10^3<br>TOF = 1.2 × 10^2 h^{-1}<br>Rate = 0.04 mM h^{-1},<br>FE_{Total} = 64%	This work
FDH/F_{Ald}DH/ADH<br>N/A<br>Cp*Rh(bpy)-mediator	MeOH	11.21 µM h^{-1},	[27]
CcFdh/PcFAlDH/YADH<br>N/A<br>Cp*Rh(bpy)-mediator	MeOH	Rate: 0.22 mM h^{-1},<br>1.28 mmol g_{pc}^{-1} h^{-1}	[28]
Photochemical			
ADH<br>N/A<br>Cp*Rh(bpy)-mediator	Butanol	TON: 6.7 4 × 10^2<br>TOF= 1.3 × 10^3 h^{-1}<br>internal quantum yield (IQY) of 0.17%.	[29]
TsOYE<br>N/A<br>Cp*Rh(bpy)-mediator	(R)-2-methylcyclohexan-1-one	TOF 4.0 × 10^2 h-1<br>Yield 59%<br>Ee 94%	[30]
YADH<br>N/A<br>Cp*Rh(bpy)-mediator<br>Via DTS/Rh@CMPs	MeOH	Rate: 0.45 mM h^{-1}	[31]
ADH<br>COF@Rh|Si<br>Cp*Rh(bpy)-mediator	Butanol	Rate 2.2 mM h^{-1}	[32]

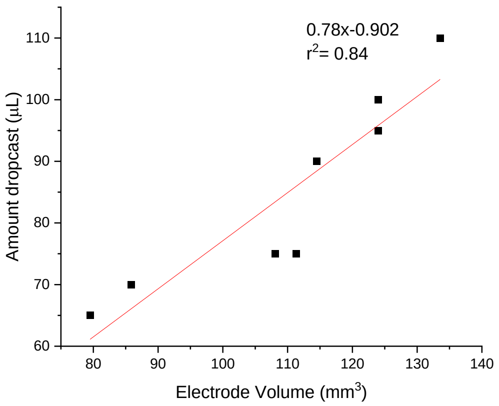

Figure S1. Estimation of the amount of ITO NPs (y-axes) to saturate the CF cuboids of different volumes (x-axes, thickness: 3.18 mm^3) by varying the size of the CF. The CF electrode was saturated with a dispersion of ITO NPs (40 nm, 100 mg mL^{-1} in 5:1 MeOH:AcOH). The point of saturation was reached as soon as the dispersion was no longer absorbed by the CF judged by eye. An ITO loading of ~6 mg on a standard 5×5×3.18 mm^3 electrode was used throughout the electrochemical and photoelectrochemical experiments.

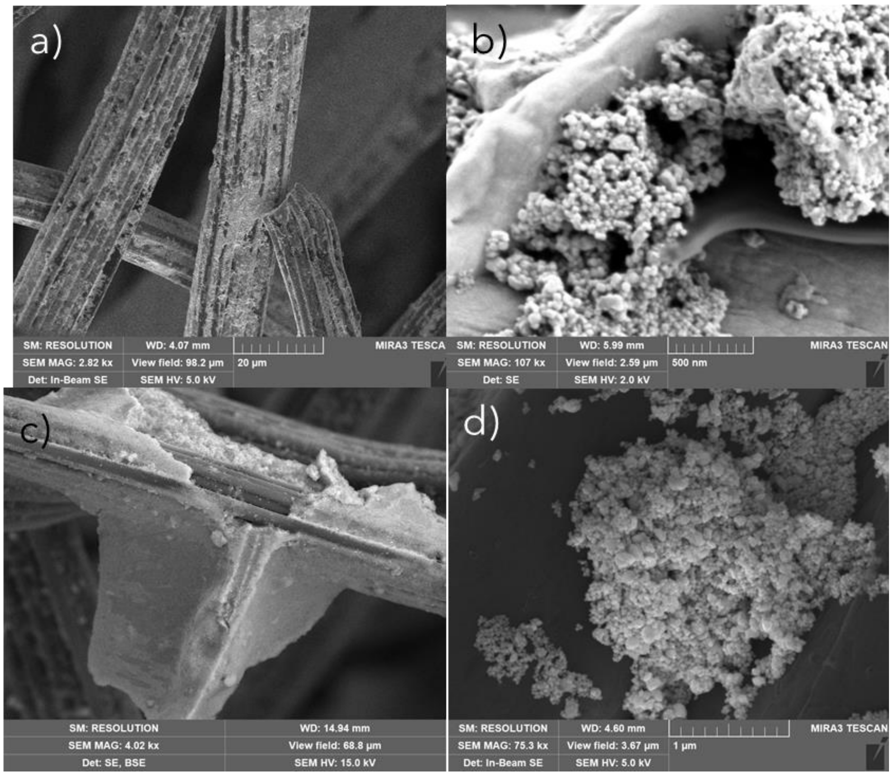

Figure S2. SEM images of ITO-coated carbon felt electrodes (ITO-CF). The ITO NPs are visible on the CF fibers. (a–d) represent different areas of the ITO-CF electrode at different zooms (indicated in the figure).

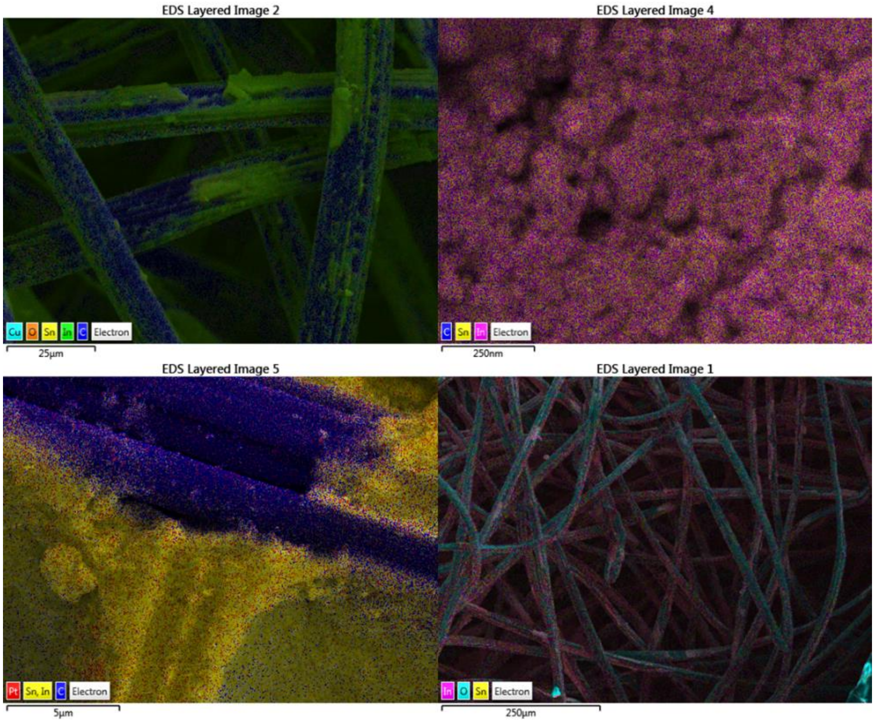

Figure S3. EDX spectroscopy of ITO-CF electrodes, showing the mesoporous ITO layer and the underlying carbon felt substrate. The ITO NPs are visible on the CF fibers, demonstrated by the presence of In, Sn and O elements.

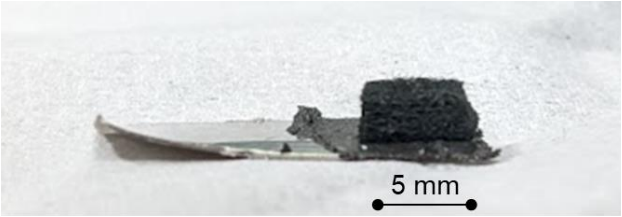

Figure S4. Photographic picture of a stationary Ti|ITO-CF electrode used in the 2-compartment 15 mL electrochemical cell.

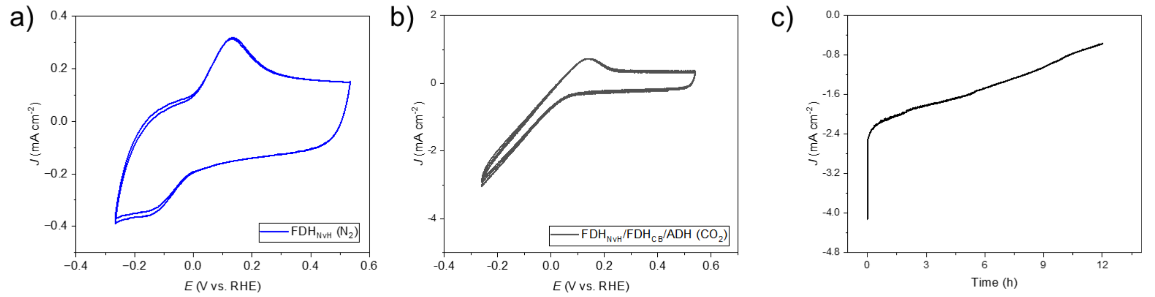

Figure S5. Electrochemical characterization of Ti|ITO-CF|FDH_{NvH} and the cascade Ti|ITO-CF|FDH_{NvH}/FDH_{CB}/ADH employing 15 mL electrolyte. a) PFV recorded at 10 mV s^{-1} for Ti|ITO-CF|FDH_{NvH} in presence of N_2 and in absence of CO_2, showing a non-turnover response. b) PFV with ITO-CF|FDH_{NvH}/FDH_{CB}/ADH recorded at 10 mV s^{-1} under CO_2 atmosphere, and c) PF-CA with ITO-CF|FDH_{NvH}/FDH_{CB}/ADH at –0.25 V vs. RHE for 12 h under CO_2 atmosphere. ^{1}H NMR spectroscopy was used to quantify the product (22 µmol of 1-phenylethanol produced after 12 h; estimated TON_{ADH} = 5.0 × 10^3).

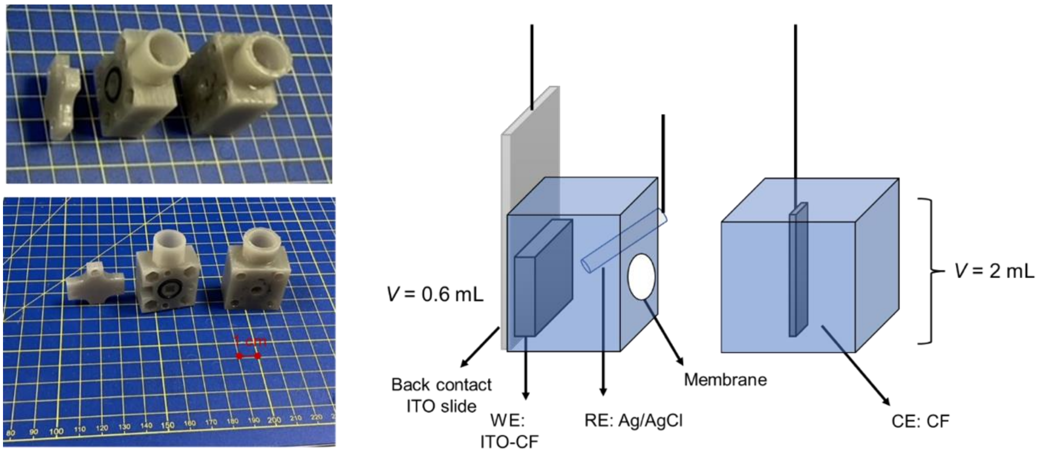

Figure S6. Photographic images, design and schematic representation of the small (0.6 mL) electrochemical cell. This small cell was developed to improve the determination of the enantiomeric excess (*ee*). To study the cascade catalysis in a small electrolyte volume, a custom cathode compartment was designed using Autodesk Fusion 360, consisting of a working compartment and counter compartment. The compartments were printed using low-force stereolithography (Formlabs 3B+, Formlabs Tough 1500 Resin). The ITO-CF cuboid was connected by pressing an ITO glass plate (8 mm × 20 mm) to the felt using a 3-D printed piece. Copper tape was applied to the ITO plate for better conductivity. The cell was assembled with PTFE and Viton gaskets along with a bipolar membrane). M4 × 45 mm bolts were used to clamp the two cell halves together. M4 × 15 mm hex head set screws were used to mount the two compartments.

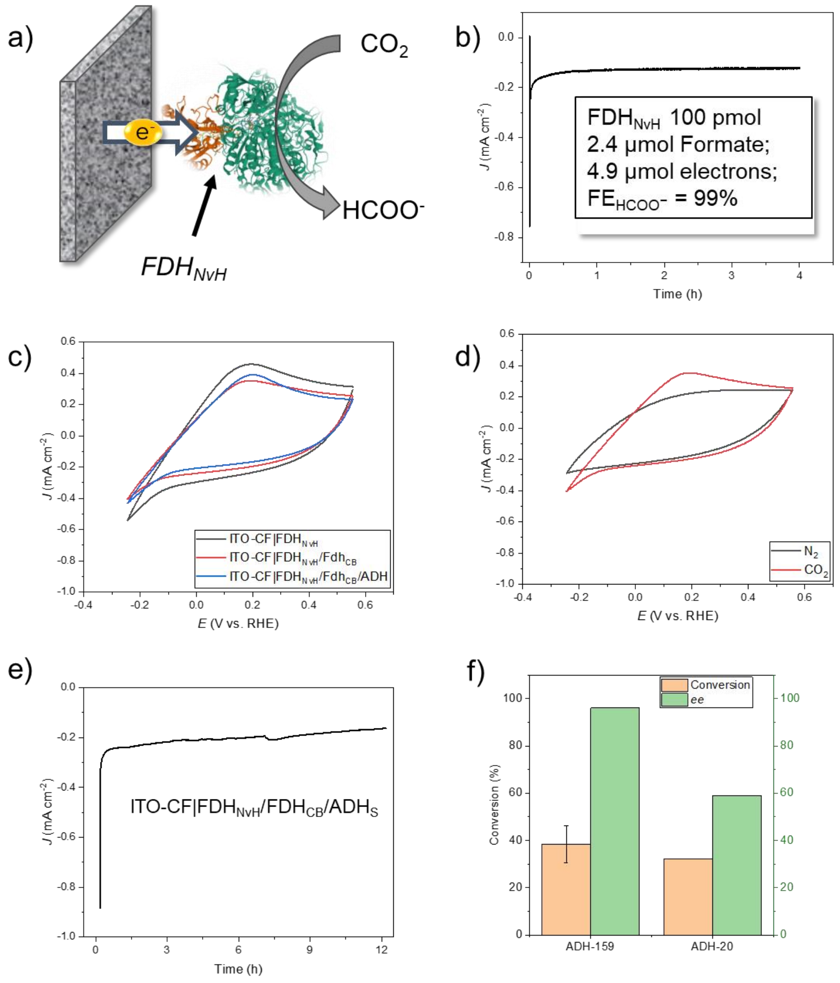

Figure S7. Electrochemical characterization carried out in the 3-D printed small cell (0.6 mL electrolyte) employing the ITO-CF cuboid electrodes (Figure S6). a) Schematic representation of the FDH_{NvH} dropcast onto the on ITO-CF|FDH_{NvH} electrode generating $HCOO^-$. b) PF-CA of FDH_{NvH}, in electrolyte saturated with CO_2 (see Table S1) where 2.4 µmol $HCOO^-$ were generated after 4 hours with quantitative FE (FE_{HCOO^-} = 99%). c) PFV scans recorded in presence of CO_2 with FDH_{NvH} (black), FDH_{NvH}/FDH_{CB} (red) and FDH_{NvH}/FDH_{CB}/ADH (blue). d) PFVs recorded in absence of CO_2 (N_2, black) and presence of CO_2 using ITO-CF|FDH_{NvH}/FDH_{CB}/ADH. e) PF-CA at –0.25 V vs. RHE employing ITO-CF|FDH_{NvH}/FDH_{CB}/ADH during 12 h in presence of CO_2. f) Product distribution of the conversion of acetophenone into (*S*)-1-phenylethanol and (*R*)-1-phenylethanol including *ee* after chronoamperometry at –0.25V for 12 h in presence of CO_2. Product quantification was performed with chiral HPLC and ^1H NMR spectroscopy.

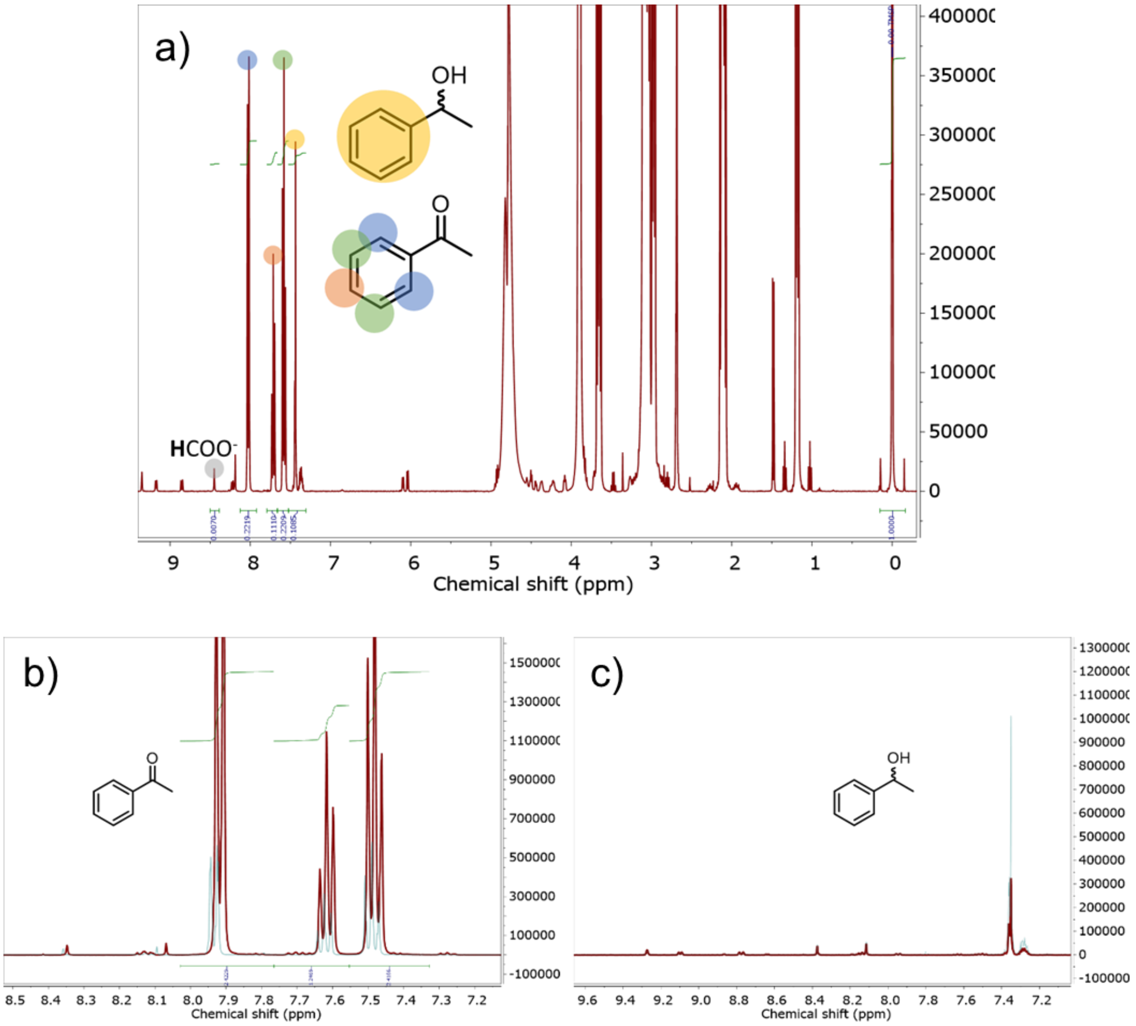

Figure S8. a) Example ^{1}H NMR spectrum after PF-CA at –0.25 V vs. RHE after 12 h with ITO-CF|FDH_{NvH}/FDH_{CB}/ADH using a small cell (V=0.6 mL) using a water suppression pulse programme. Quantification with TMSP as internal standard at 0 ppm to determine the FE_{HCOO^-}, the conversion and TON_{ADH}. ^{1}H NMR spectra to indicate the leakage of the organic molecules into the *Formlabs Tough 1500 Resin* of the 3-D printed cell, hampering the determination for the FE_{PE}. Only the conversion of acetophenone (starting from 50 mM) and FE_{HCOO^-} could be determined for the PF-CA inside the small compartment cell. b) acetophenone (50 mM at t = 0 in red and after 12 h in blue) and c) 1-phenylethanol after 12 h (at t = 0 in red and after 12 h in blue). Quantification with TMSP as internal standard showed a 4.5 × decrease in acetophenone and of 1-phenylethanol after the electrolyte remained in the cell for 12 h. This leakage was consistent throughout all experiments. The generated $HCOO^-$ is not leaking inside the resin of the 3-D printed cell.

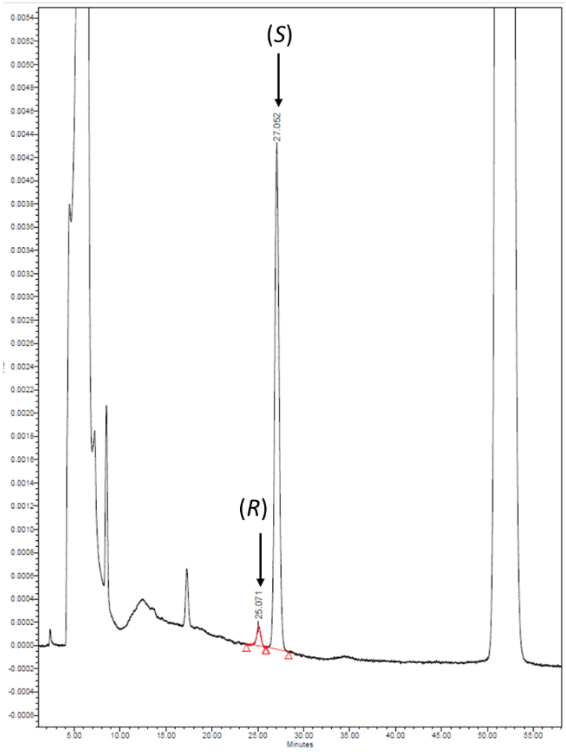

	Name	Retention Time (min)	Area (µV*sec)	% Area	Height (µV)	Int Type	Amount	Units	Peak Type	Peak Codes
1		25.071	5026	3.32	160	bb			Unknown	
2		27.052	146343	96.68	4319	bb			Unknown	

Figure S9. HPLC trace of (*S*)-1-phenylethanol produced by Ti|ITO-CF|FDH_{NvH}/FDH_{CB}/ADH_{S} electrode.

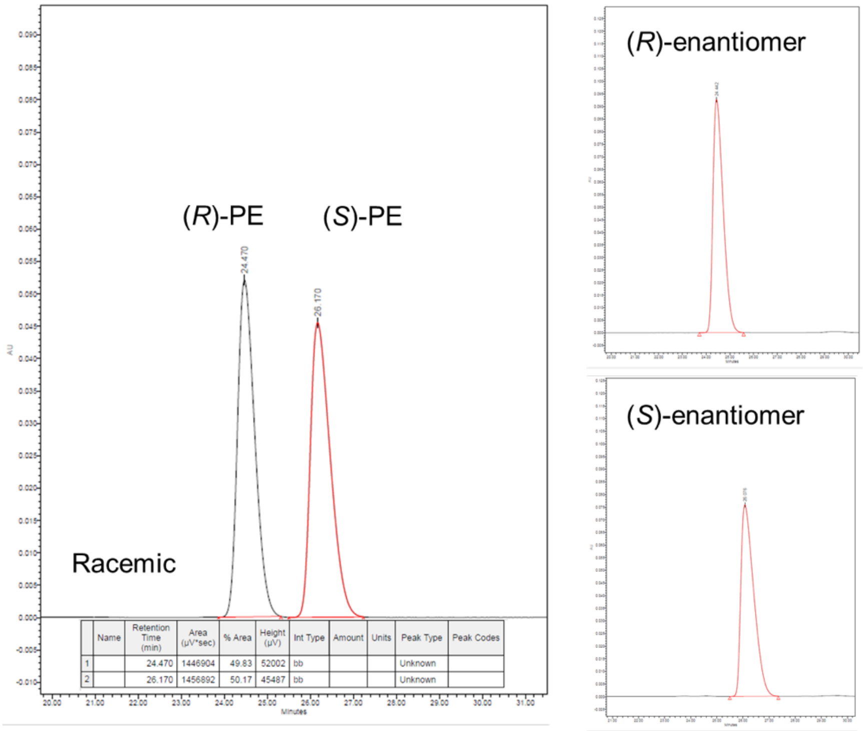

	Name	Retention Time (min)	Area (µV*sec)	% Area	Height (µV)	Int Type	Amount	Units	Peak Type	Peak Codes
1		24.470	1446904	49.83	52002	bb			Unknown	
2		26.170	1456892	50.17	45487	bb			Unknown	

Figure S10. HPLC of commercial 1-phenylethanol. Racemic, (*R*)-1-phenylethanol than (*S*)-1-phenylethanol.

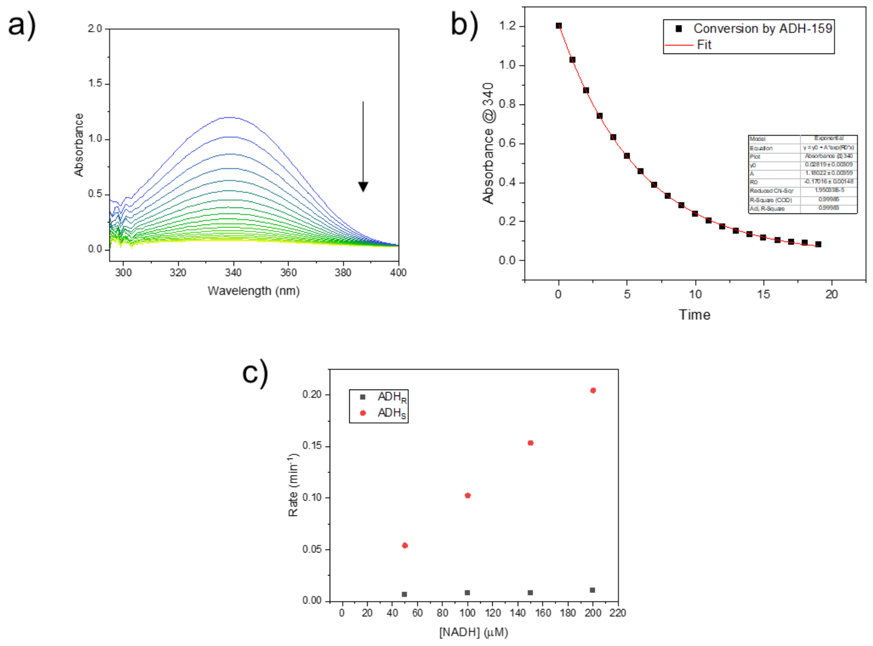

Figure S11. Kinetics of consumption of NADH by ADH_S and ADH_R. ADH (500 µg) was dissolved into the reaction mixture (2 mL, 0.1 M MOPS, pH 7) consisting of acetophenone, (50 mM) and HCOONa (200 mM). a) The absorbance of 340 nm is decreasing over time due to the conversion of NADH (λ_{msx} = 340 nm, starting concentration = 200 µM) to NAD^+ by ADH_S in the course of 20 minutes. b) The absorbance at 340 nm (corresponding to the concentration of NADH in the cuvette) over the course of 20 min and the fit. This decrease in absorbance relates to the rate of the ADH_S for ketone reduction accompanied by NADH consumption c) Difference in rate of ADH_S (red dots) and ADH_R (black squares) using a different concentration of NADH (50, 100, 150 and 200 µM).

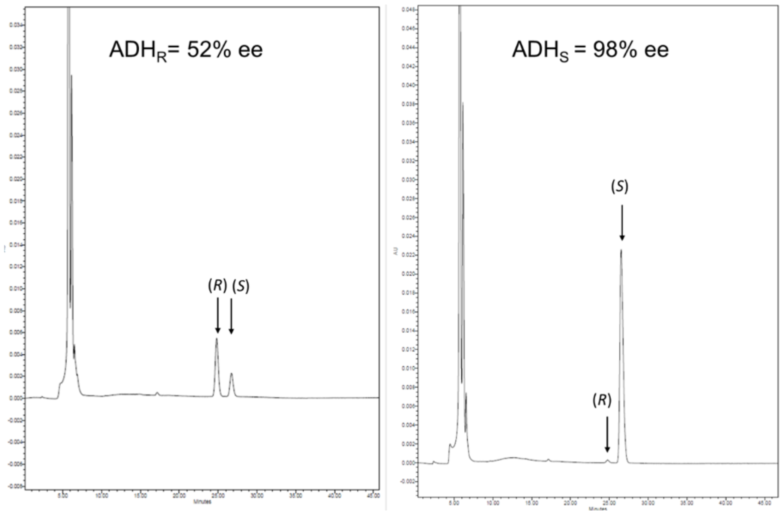

Figure S12. HPLC traces of a reaction mixture employing “free enzyme” ADH_R or ADH_S using the standard screening procedure: ADH (5 mg) was weight into an Eppendorf reaction vial. In a separate container the reaction mix was prepared by dissolving NAD^+ (6.8 mg), sodium formate (138 mg) and FDH-102 (10 mg) in MOPS buffer (450 µL, pH 7). The reaction mix (450 µL) was then added to the ADH powder. Then acetophenone solution in DMSO was added to the mixture (50 µL, 500 mM, final concentration of acetophenone in the catalysis mixture is 50 mM). The reaction was incubated for 18 hours at 30 °C. The relative amounts for (R) or (S)-1-phenylethanol were measured with chiral HPLC.

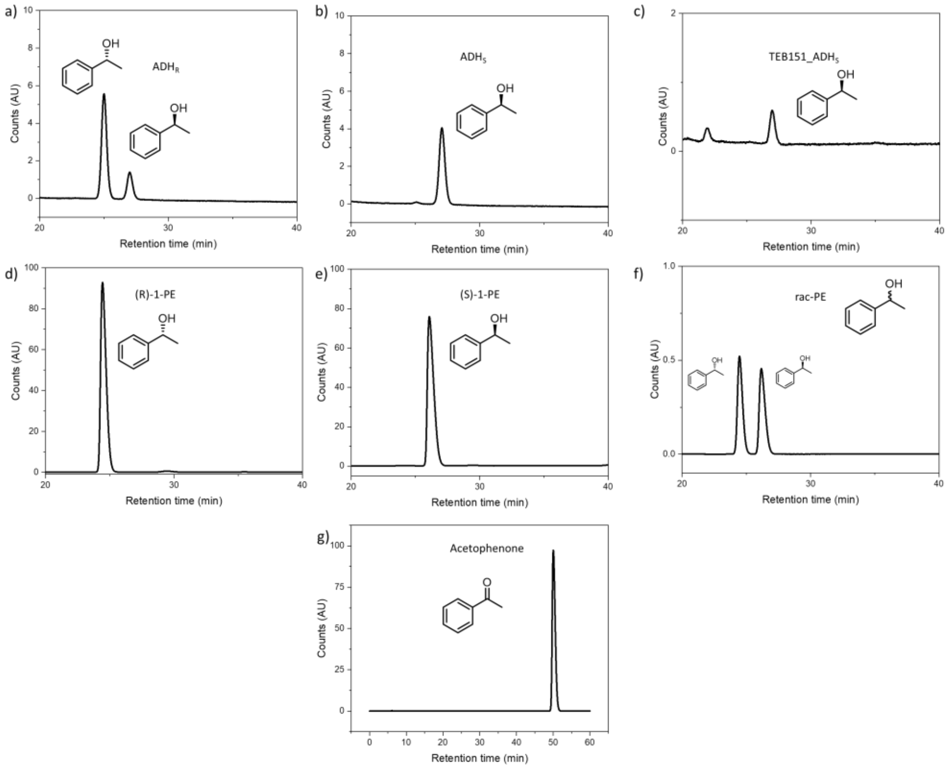

Figure S13. Processed HPLC data for PF-CA with ITO-CF|FDH$_{NvH}$/FDH$_{CB}$/ADH employing a) ADH$_R$ (using 0.6 mL electrolyte) and b) ADH$_S$ (using 0.6 mL electrolyte). c) photoelectrochemical data employing OPV|ITO-CF|FDH$_{NvH}$/FDH$_{CB}$/ADH (using 15 mL electrolyte). commercially available d) (*R*)-1-phenylethanol, e) (*S*)-1-phenylethanol f) racemic 1-phenylethanol, g) acetophenone.

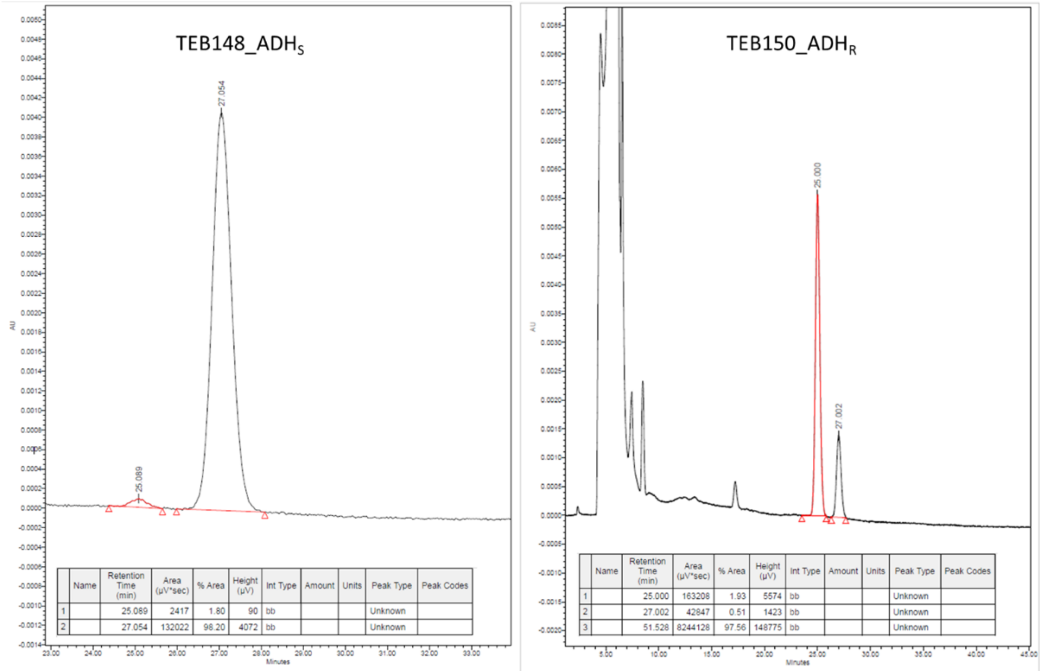

	Name	Retention Time (min)	Area (µV*sec)	% Area	Height (µV)	Int Type	Amount	Units	Peak Type	Peak Codes
1		25.089	2417	1.80	90	bb			Unknown	
2		27.054	132022	98.20	4072	bb			Unknown	

	Name	Retention Time (min)	Area (µV*sec)	% Area	Height (µV)	Int Type	Amount	Units	Peak Type	Peak Codes
1		25.000	163208	1.93	5574	bb			Unknown	
2		27.002	42847	0.51	1423	bb			Unknown	
3		51.528	8244128	97.56	148775	bb			Unknown	

Figure S14. HPLC after PF-CA (electrolysis) using ITO-CF|FDH$_{NvH}$/FDH$_{CB}$/ADH for 12 hours (0.6 mL cell), where ADH$_S$ is generating (S)-1-phenylethanol and ADH$_R$ is generating more (R)-1-phenylethanol than (S)-1-phenylethanol.

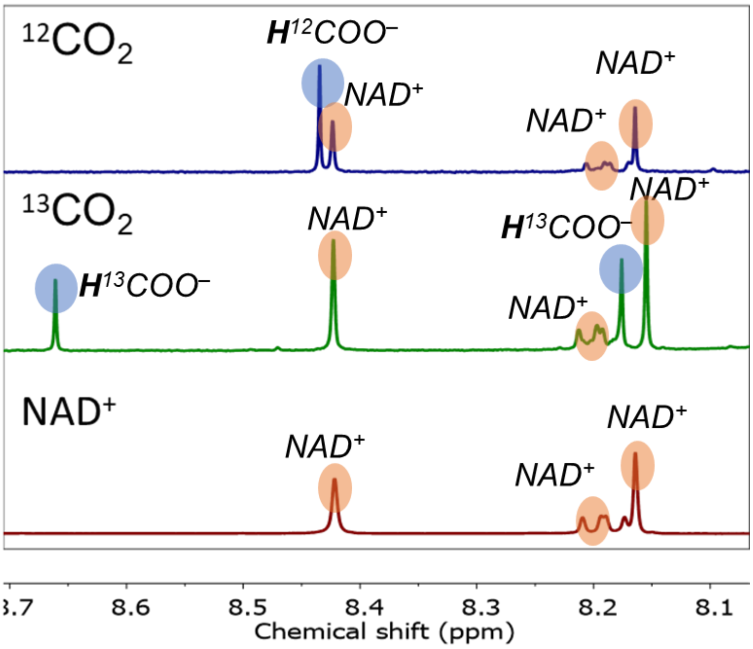

Figure S15. Isotopic labeling experiment using $^{13}CO_2$ vs. $^{12}CO_2$ to demonstrate that the generated $HCOO^-$ originates from CO_2-reduction. ^1H NMR spectra after the PF-CA experiment using ITO-CF|FDH$_{NvH}$/FDH$_{CB}$/ADH in 0.6 mL electrolyte for 4 hours of $^{12}CO_2$ (upper spectrum blue line) vs. $^{13}CO_2$ (middle spectrum, green line), confirms that $HCOO^-$ solely originates from CO_2 (^{1}H-NMR, doublet, $J_{C\text{–}H}$ = 194 Hz). The ^{1}H NMR resonance of $H^{12}COO^-$ (blue circles) and $H^{13}COO^-$ (blue circles) and NAD$^+$ (red circles) partly overlap. Therefore, the ^{1}H NMR of NAD$^+$ is depicted for comparison (red spectrum).

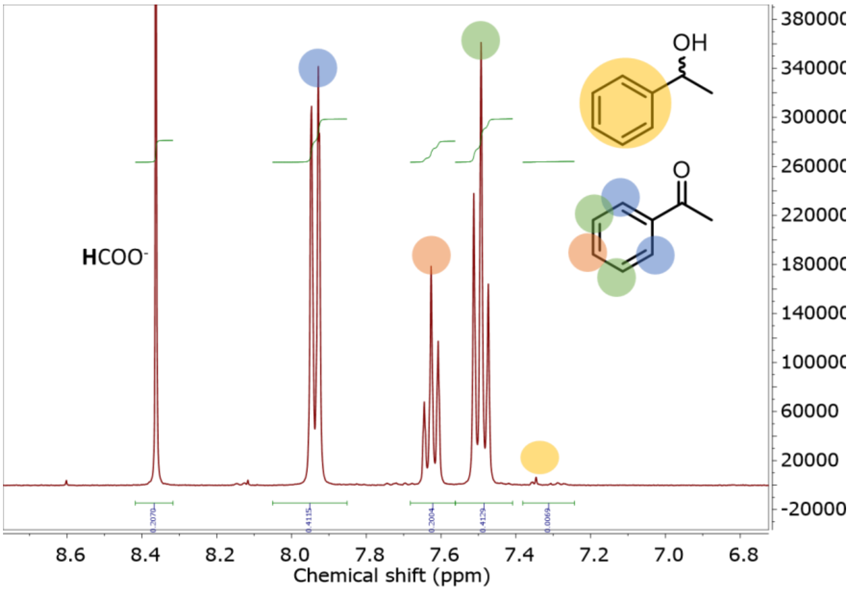

Figure S16. Control experiment for PF-CA using ITO-CF|FDH$_{NvH}$/FDH$_{CB}$/ADH with without NAD$^+$ using 0.6 mL electrolyte. The ADH used in this study is a lysate and verify the potential presence of NAD$^+$ or NADH, we performed a control experiment in the absence of additional NAD$^+$ in the electrolyte solution. We observed only marginal conversion of acetophenone into 1-phenylethanol (< 1%) at –0.25 V vs. RHE after 12 hours. After PF-CA in the presence of NAD$^+$ in the electrolyte, the conversion of acetophenone into 1-phenylethanol is 38±8%.

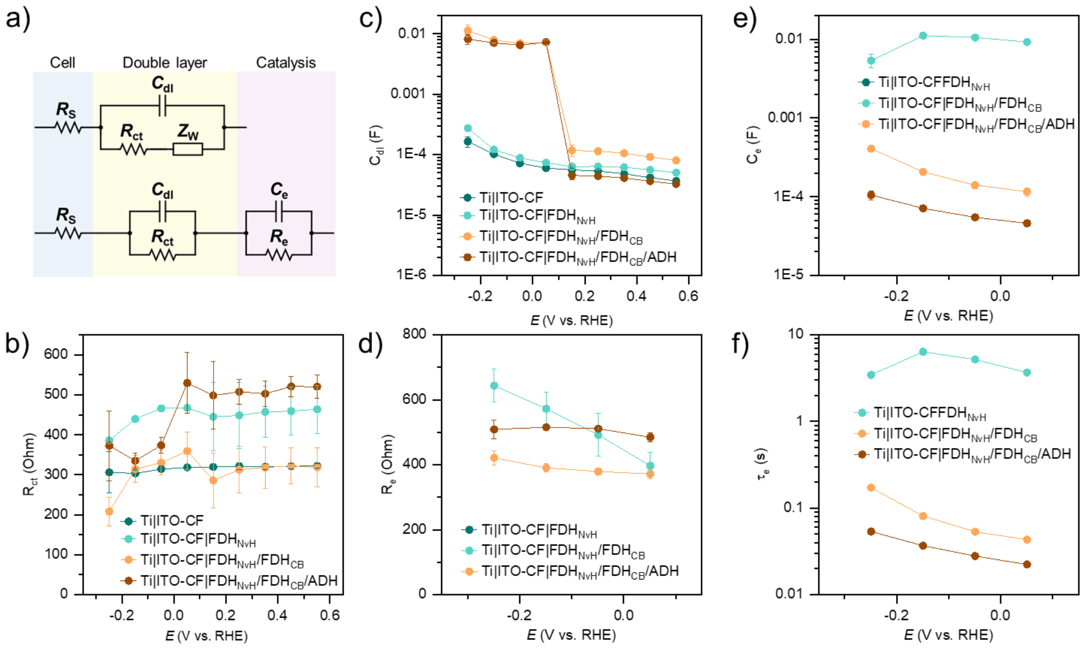

Figure S17. EIS analysis for Ti|ITO-CF, Ti|ITO-CF|FDH$_{NvH}$, Ti|ITO-CF|FDH$_{NvH}$/FDH$_{CB}$ and Ti|ITO-CF|FDH$_{NvH}$/FDH$_{CB}$/ADH in 15 mL electrolyte solution. a) Equivalent circuit used to fit the EIS data. b) The charge transfer resistance (R_{CT}) at different applied potentials. c) The diffusion layer (C_{dl}) d) The electrocatalysis resistance (R_e) and e) electrocatalysis capacitance (C_e) at different applied potentials. f) The electrocatalysis time constant (τ_e) at different applied potentials.

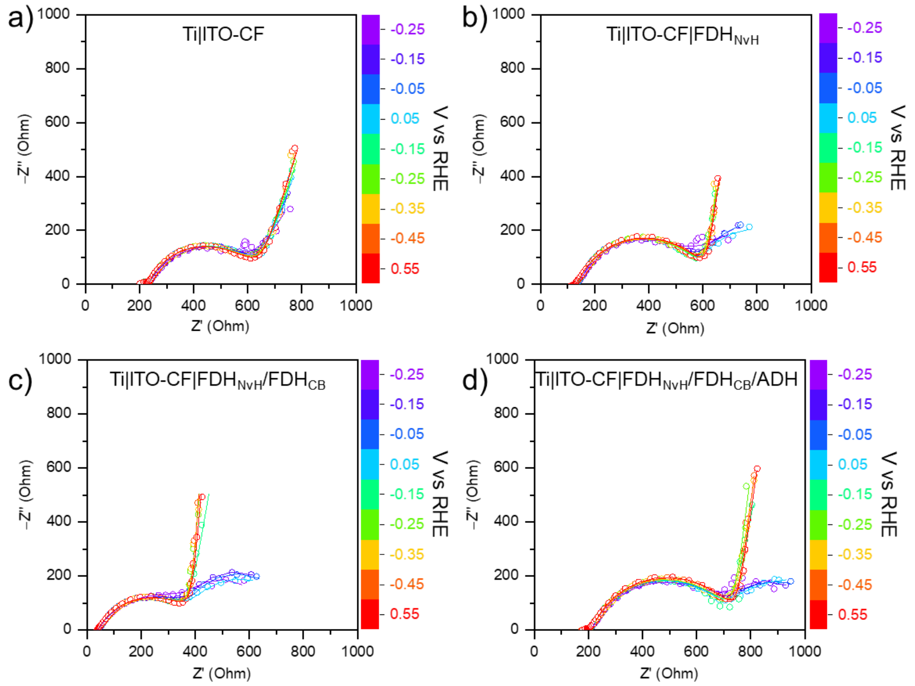

Figure S18. Nyquist plots with corresponding fitting curves for a) Ti|ITO-CF, b) Ti|ITO-CF|FDH$_{NvH}$, c) Ti|ITO-CF|FDH$_{NvH}$/Fdh and d) Ti|ITO-CF|FDH$_{NvH}$/FDH$_{CB}$/ADH. EIS recorded in 15 mL electrolyte solution.

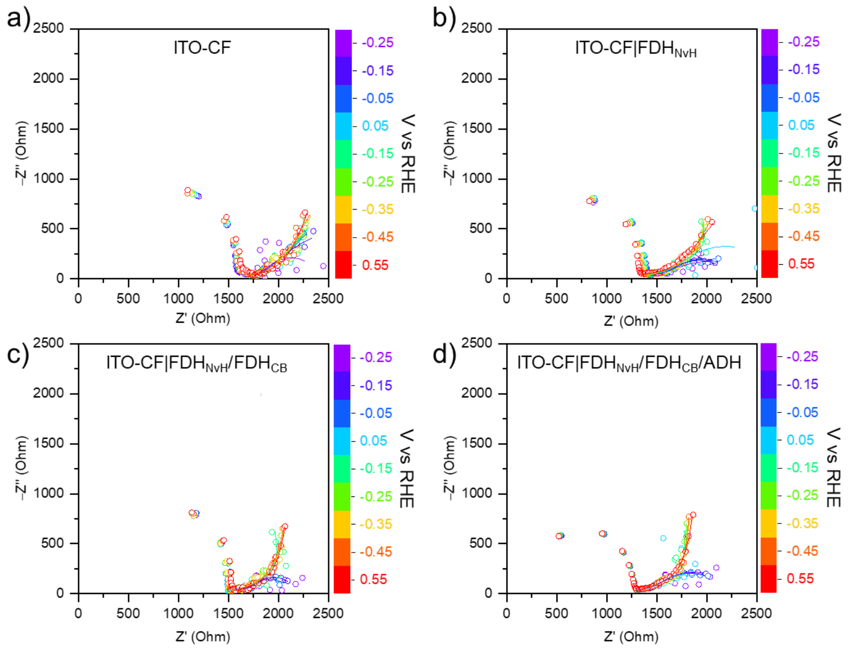

Figure S19. Nyquist plots with corresponding fitting curves using the ITO-CF cuboids for a) ITO-CF b) ITO-CF|FDH_{NvH}, c) ITO-CF|FDH_{NvH}/FDH_{CB} and d) ITO-CF|FDH_{NvH}/FDH_{CB}/ADH. EIS recorded in 0.6 mL electrolyte solution.

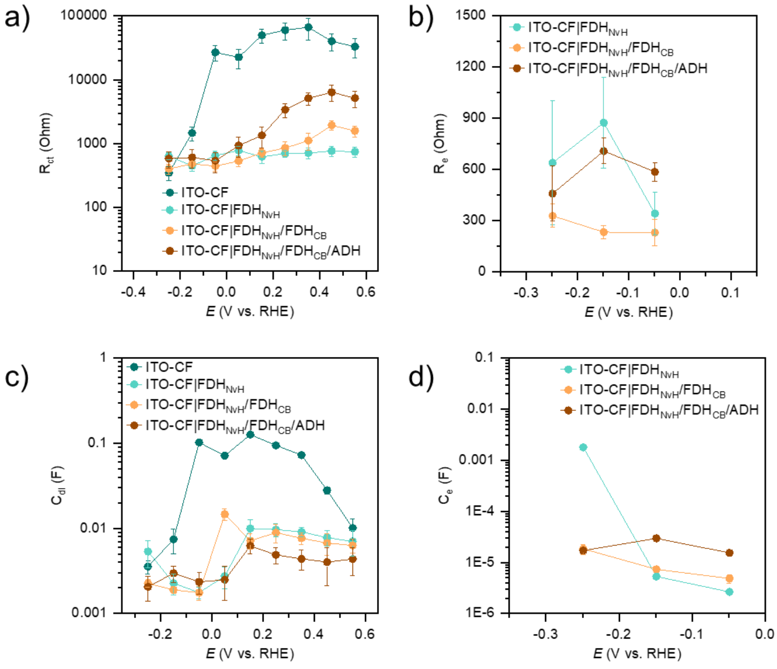

Figure S20. EIS fitting results for in the 0.6 mL electrolyte in the 3-D printed cell using the ITO-CF cuboids analyzing ITO-CF, ITO-CF|FDH_{NvH}, ITO-CF|FDH_{NvH}/FDH_{CB} and ITO-CF|FDH_{NvH}/FDH_{CB}/ADH electrodes. a) The charge transfer resistance (R_{CT}) different applied potentials. b) The electrocatalysis resistance (R_e) an c) The diffusion layer capacitance (C_{dl}) d) eletrocatalysis capacitance (C_e) at different applied potentials.

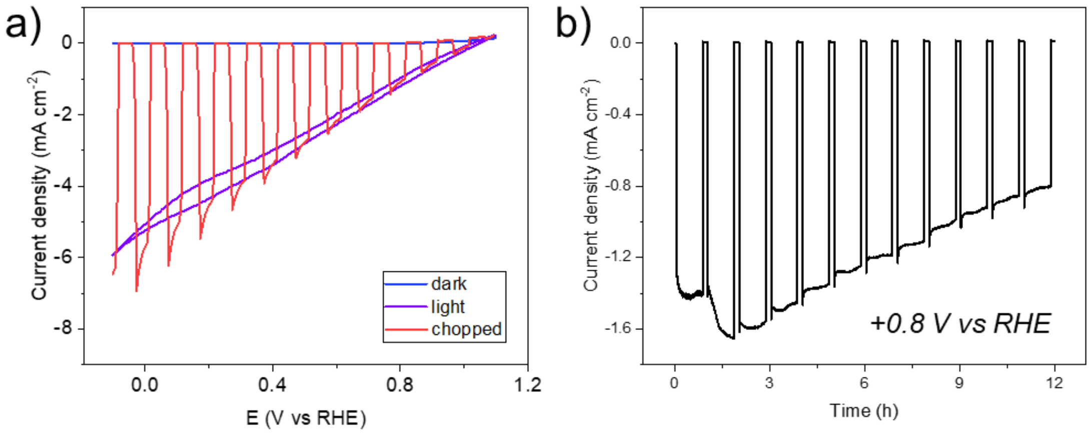

Figure S21. Repeat of the OPV-experiment discussed in the main text (Figure 3), employing OPV|ITO-CF|FDH_{NvH}/FDH_{CB}/ADH for light driven ketone reduction using 15 mL electrolyte a) PFV scans in dark, light and chopped with on/off cycles of 10 seconds. b) Chopped light CA at +0.8 V vs. RHE with 50 min on and 10 min off.

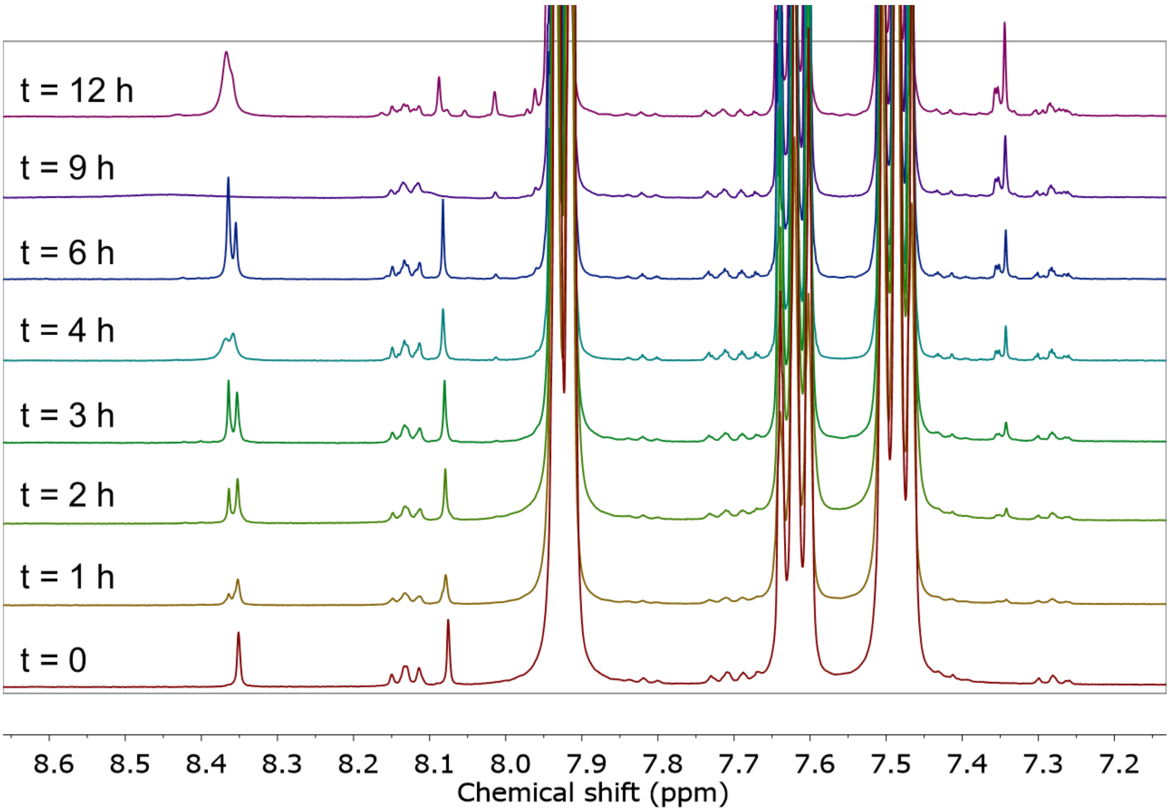

Figure S22. Product formation during PEC experiment using 15 mL electrolyte. ^{1}H NMR spectra recorded for chopped light PF-CA with OPV|ITO-CF| FDH_{NvH} /FDH_{CB}/ADH at -0.25 V vs. RHE with 50 min on and 10 min off. NMR samples were collected after t=0, 1, 2, 3, 4, 6, 9 and 12 h. We observed the formation of 1-phenylethanol (5.3 µmol) after 12 h and estimated the TON_{ADH} to be 12×10^{2}.

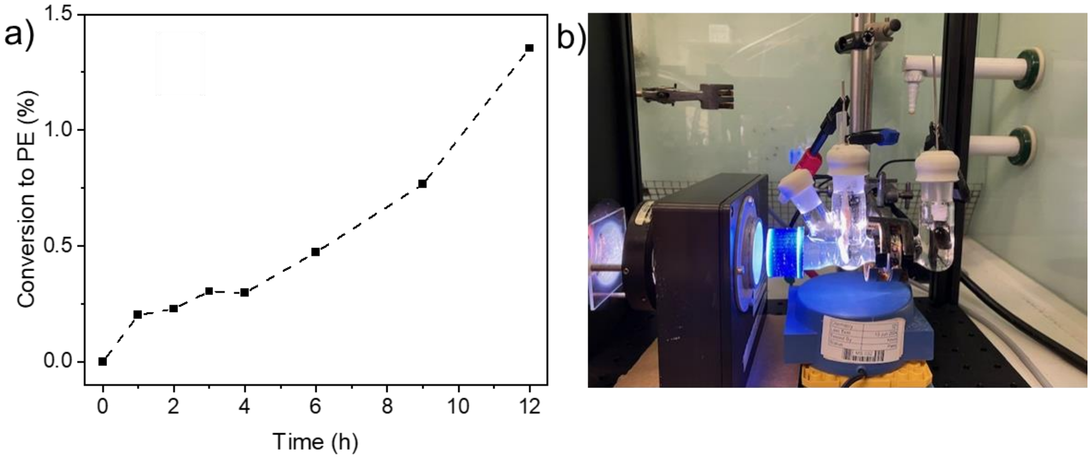

Figure S23. Production of (S)-1-phenylethanol (PE) using a OPV|ITO-CF|FDH_{NvH}/FDH_{CB}/ADH photocathode (PF-CA at +0.8 V vs. RHE with 50 min on and 10 min off, using 15 mL electrolyte). a) NMR samples were collected after t = 0, 1, 2, 3, 4, 6, 9 and 12 h and the conversion of acetophenone to phenylethanol (PE) is plotted against time. The conversion is compensated for the decrease in electrolyte volume, as 0.5 mL per sample is removed from the bulk. b) A photo of the PEC experiment in showing the 2-compartment cell (left = working compartment, cathodic chamber; right = counter compartment, anodic chamber).

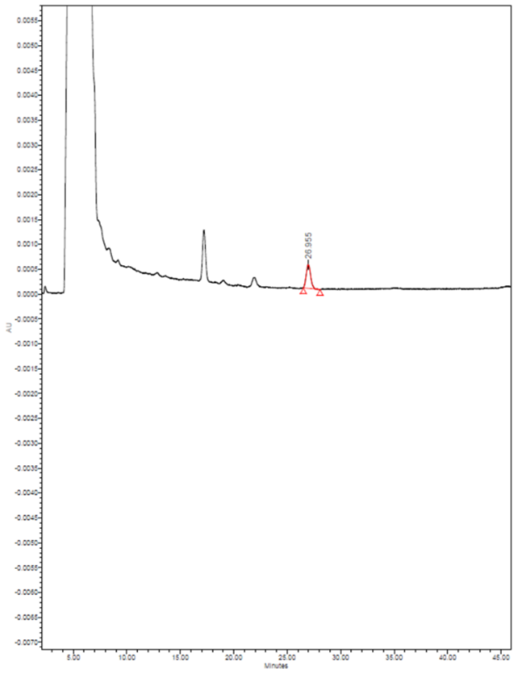

Figure S24. HPLC of (*S*)-1-phenylethanol produced by OPV|ITO-CF|FDH_{NvH}/FDH_{CB}/ADH photocathode using 15 mL electrolyte.

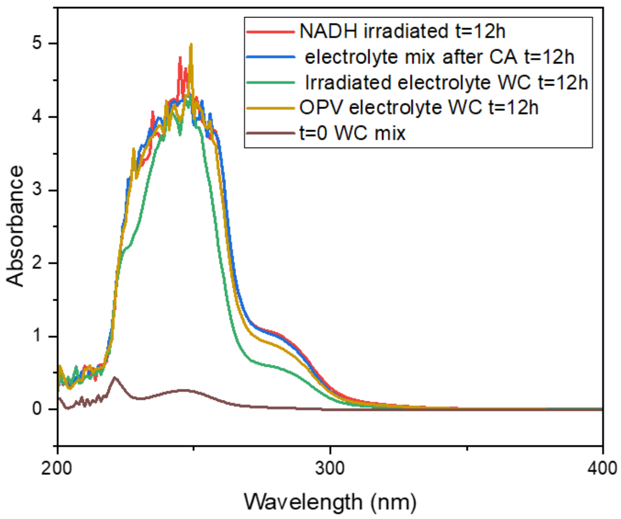

Figure S25. UV–Vis absorption spectra to study the degradation of NADH (using 15 mL electrolyte). Red: absorption spectrum of NADH (1 mM, 15 mL) after 12 hours of stirring under AM1.5G irradiation (no voltage applied). Blue: absorption spectrum of the working compartment electrolyte containing NADH (1 mM, 15 mL) after 12 hours of chronoamperometry (not irradiated) at –0.25 V vs. RHE. Green: absorption spectrum of the electrolyte after 12 hours AM1.5G irradiation experiment (no potential). Yellow: absorption spectrum of the electrolyte after 12 hours AM1.5G irradiation after the PEC experiment at 0.8 V vs. RHE. Brown: the electrolyte mix before the irradiation experiment at t = 0. The absorption at 280 nm and the absence of absorption at 340 nm indicates that NADH has degraded after 12 hours AM1.5G irradiation.

Supporting References

(1) Oliveira, A. R.; Mota, C.; Vilela-Alves, G.; Manuel, R. R.; Pedrosa, N.; Fourmond, V.; Klymanska, K.; Léger, C.; Guigliarelli, B.; Romão, M. J.; Cardoso Pereira, I. A. An Allosteric Redox Switch Involved in Oxygen Protection in a CO2 Reductase. *Nat. Chem. Biol.* **2024**, *20* (1), 111–119. https://doi.org/10.1038/s41589-023-01484-2.

(2) Cobb, S. J.; Dharani, A. M.; Oliveira, A. R.; Pereira, I. A. C.; Reisner, E. Carboxysome-Inspired Electrocatalysis Using Enzymes for the Reduction of CO_2 at Low Concentrations. *Angew. Chem. Int. Ed.* **2023**, *62* (26) No. e202218782. https://doi.org/10.1002/anie.202218782.

(3) Andrei, V.; Reuillard, B.; Reisner, E. Bias-Free Solar Syngas Production by Integrating a Molecular Cobalt Catalyst with Perovskite–BiVO4 Tandems. *Nat. Mater.* **2020**, *19* (2), 189–194. https://doi.org/10.1038/s41563-019-0501-6.

(4) Andrei, V.; Jagt, R. A.; Rahaman, M.; Lari, L.; Lazarov, V. K.; MacManus-Driscoll, J. L.; Hoye, R. L. Z.; Reisner, E. Long-Term Solar Water and CO2 Splitting with Photoelectrochemical BiOI–BiVO4 Tandems. *Nat. Mater.* **2022**, *21* (8), 864–868. https://doi.org/10.1038/s41563-022-01262-w.

(5) In this study, the ADH enzymes have a molecular weight of approximately 27 kDa and the wt% is between 20–30% of total cell protein. We calculated that 0.5 mg of ADH translates into 4–6 nmol and throughout this work, we report 5 nmol.

(6) Yeung, C. W. S.; Andrei, V.; Lee, T. H.; Durrant, J. R.; Reisner, E. Organic Semiconductor-$BiVO_4$ Tandem Devices for Solar-Driven H_2O and CO_2 Splitting. *Adv. Mater.* **2024**, *36* (35) 2404110. https://doi.org/10.1002/adma.202404110.

(7) Liu, Y.; Pulignani, C.; Webb, S.; Cobb, S. J.; Rodríguez-Jiménez, S.; Kim, D.; Milton, R. D.; Reisner, E. Electrostatic [FeFe]-Hydrogenase–Carbon Nitride Assemblies for Efficient Solar Hydrogen Production. *Chem. Sci.* **2024**, *15* (16), 6088–6094. https://doi.org/10.1039/D4SC00640B.

(8) Liu, Y.; Bin Mohamad Annuar, A.; Rodríguez-Jiménez, S.; Yeung, C. W. S.; Wang, Q.; Coito, A. M.; Manuel, R. R.; Pereira, I. A. C.; Reisner, E. Solar Fuel Synthesis Using a Semiartificial Colloidal Z-Scheme. *J. Am. Chem. Soc.* **2024**, *146* (43), 29865–29876. https://doi.org/10.1021/jacs.4c11827.

(9) Yeung, C. W. S.; Liu, Y.; Cobb, S.; Andrei, V.; Coito, A.; Manuel, R.; Pereira, I.; Reisner, E. Semi-Artificial Leaf Interfacing Organic Semiconductors and Enzymes for Solar Fuel Synthesis. December 20, 2024. https://doi.org/10.26434/chemrxiv-2024-f49zl.

(10) Cobb, S. J.; Badiani, V. M.; Dharani, A. M.; Wagner, A.; Zacarias, S.; Oliveira, A. R.; Pereira, I. A. C.; Reisner, E. Fast CO2 Hydration Kinetics Impair Heterogeneous but Improve Enzymatic CO2 Reduction Catalysis. *Nat. Chem.* **2022**, *14* (4), 417–424. https://doi.org/10.1038/s41557-021-00880-2.

(11) Edwardes Moore, E.; Cobb, S. J.; Coito, A. M.; Oliveira, A. R.; Pereira, I. A. C.; Reisner, E. Understanding the Local Chemical Environment of Bioelectrocatalysis. *Proc. Natl. Acad. Sci. U. S. A.* **2022**, *119* (4) No. e2114097119. https://doi.org/10.1073/pnas.2114097119.

(12) Pandey, K.; Islam, S. T. A.; Happe, T.; Armstrong, F. A. Frequency and Potential Dependence of Reversible Electrocatalytic Hydrogen Interconversion by [FeFe]-Hydrogenases. *Proc. Natl. Acad. Sci. U. S. A.* **2017**, *114* (15), 3843–3848. https://doi.org/10.1073/pnas.1619961114.

(13) Randles, J. E. B. Kinetics of Rapid Electrode Reactions. *Discuss. Faraday Soc.* **1947**, *1*, 11–19. https://doi.org/10.1039/df9470100011.

(14) Liu, Y.; Xia, M.; Ren, D.; Nussbaum, S.; Yum, J.-H.; Grätzel, M.; Guijarro, N.; Sivula, K. Photoelectrochemical CO_2 Reduction at a Direct $CuInGaS_2$ /Electrolyte Junction. *ACS Energy Lett.* **2023**, *8* (4), 1645–1651. https://doi.org/10.1021/acsenergylett.3c00022.

(15) Liu, Y.; Bouri, M.; Yao, L.; Xia, M.; Mensi, M.; Grätzel, M.; Sivula, K.; Aschauer, U.; Guijarro, N. Identifying Reactive Sites and Surface Traps in Chalcopyrite Photocathodes. *Angew. Chem. Int. Ed.* **2021**, *60* (44), 23651–23655. https://doi.org/10.1002/anie.202108994.

(16) Liu, Y.; Webb, S.; Moreno-García, P.; Kulkarni, A.; Maroni, P.; Broekmann, P.; Milton, R. D. Facile Functionalization of Carbon Electrodes for Efficient Electroenzymatic Hydrogen Production. *JACS Au* **2023**, *3* (1), 124–130. https://doi.org/10.1021/jacsau.2c00551.

(17) Bisquert, J. Theory of the Impedance of Electron Diffusion and Recombination in a Thin Layer. *J. Phys. Chem. B,* **2002**, *106* (2), 325–333. https://doi.org/10.1021/jp011941g.

(18) Wieczorek, A.; Liu, Y.; Cho, H.-H.; Sivula, K. Assessing the Charge Carrier Dynamics at Hybrid Interfaces of Organic Photoanodes for Solar Fuels. *J. Phys. Chem. Lett.* **2024**, *15* (24), 6347–6354. https://doi.org/10.1021/acs.jpclett.4c01170.

(19) Cheng, B.; Wan, L.; Armstrong, F. A. Progress in Scaling up and Streamlining a Nanoconfined, Enzyme-Catalyzed Electrochemical Nicotinamide Recycling System for Biocatalytic Synthesis. *ChemElectroChem* **2020**, *7* (22), 4672–4678. https://doi.org/10.1002/celc.202001166.

(20) Siritanaratkul, B.; Megarity, C. F.; Roberts, T. G.; Samuels, T. O. M.; Winkler, M.; Warner, J. H.; Happe, T.; Armstrong, F. A. Transfer of Photosynthetic NADP+/NADPH Recycling Activity to a Porous Metal Oxide for Highly Specific, Electrochemically-Driven Organic Synthesis. *Chem. Sci.* **2017**, *8* (6), 4579–4586. https://doi.org/10.1039/c7sc00850c.

(21) Zhan, P.; Liu, X.; Zhang, S.; Zhu, Q.; Zhao, H.; Ren, C.; Zhang, J.; Lu, L.; Cai, D.; Qin, P. Electroenzymatic Reduction of Furfural to Furfuryl Alcohol by an Electron Mediator and Enzyme Orderly Assembled Biocathode. *ACS Appl. Mater. Interfaces* **2023**, *15* (10), 12855–12863. https://doi.org/10.1021/acsami.3c00320.

(22) Kurimoto, A.; Nasseri, S. A.; Hunt, C.; Rooney, M.; Dvorak, D. J.; LeSage, N. E.; Jansonius, R. P.; Withers, S. G.; Berlinguette, C. P. Bioelectrocatalysis with a Palladium Membrane Reactor. *Nat. Commun.* **2023**, *14* (1) 1814. https://doi.org/10.1038/s41467-023-37257-7.

(23) Bau, J. A.; Emwas, A.-H.; Nikolaienko, P.; Aljarb, A. A.; Tung, V.; Rueping, M. Mo^{3+} Hydride as the Common Origin of H2 Evolution and Selective NADH Regeneration in

Molybdenum Sulfide Electrocatalysts. *Nat. Catal.* **2022**, *5* (5), 397–404. https://doi.org/10.1038/s41929-022-00781-8.

(24) Yuan, M.; Kummer, M. J.; Milton, R. D.; Quah, T.; Minteer, S. D. Efficient NADH Regeneration by a Redox Polymer-Immobilized Enzymatic System. *ACS Catal.* **2019**, *9* (6), 5486–5495. https://doi.org/10.1021/acscatal.9b00513.

(25) Castañeda-Losada, L.; Adam, D.; Paczia, N.; Buesen, D.; Steffler, F.; Sieber, V.; Erb, T. J.; Richter, M.; Plumeré, N. Bioelectrocatalytic Cofactor Regeneration Coupled to CO2 Fixation in a Redox-Active Hydrogel for Stereoselective C−C Bond Formation. *Angew. Chem. Int. Ed.* **2021**, *60* (38), 21056–21061. https://doi.org/10.1002/anie.202103634.

(26) Wang, C.; Dong, W.; Zhang, P.; Ma, Y.; Han, Z.; Zou, Y.; Wang, W.; Li, H.; Hollmann, F.; Liu, J. Formate-Mediated Electroenzymatic Synthesis via Biological Cofactor NADH. *Angew. Chem. Int. Ed.* **2024**, *63* (41), No. e202408756. https://doi.org/10.1002/anie.202408756.

(27) Yadav, R. K.; Oh, G. H.; Park, N.-J.; Kumar, A.; Kong, K.; Baeg, J.-O. Highly Selective Solar-Driven Methanol from CO_2 by a Photocatalyst/Biocatalyst Integrated System. *J. Am. Chem. Soc.* **2014**, *136* (48), 16728–16731. https://doi.org/10.1021/ja509650r.

(28) Kuk, S. K.; Singh, R. K.; Nam, D. H.; Singh, R.; Lee, J.; Park, C. B. Photoelectrochemical Reduction of Carbon Dioxide to Methanol through a Highly Efficient Enzyme Cascade. *Angew. Chem. Int. Ed.* **2017**, *56* (14), 3827–3832. https://doi.org/10.1002/anie.201611379.

(29) Chakraborty, I. N.; Jain, V.; Roy, P.; Kumar, P.; Vinod, C. P.; Pillai, P. P. Photocatalytic Regeneration of Reactive Cofactors with InP Quantum Dots for the Continuous Chemical Synthesis. *ACS Catal.* **2024**, *14* (9), 6740–6748. https://doi.org/10.1021/acscatal.4c00817.

(30) Kim, J.; Lee, S. H.; Tieves, F.; Choi, D. S.; Hollmann, F.; Paul, C. E.; Park, C. B. Biocatalytic C=C Bond Reduction through Carbon Nanodot-Sensitized Regeneration of NADH Analogues. *Angew. Chem. Int. Ed.* **2018**, *57* (42), 13825–13828. https://doi.org/10.1002/anie.201804409.

(31) Wu, X.; Wang, S.; Fang, J.; Chen, H.; Liu, H.; Li, R. Enhanced Photocatalytic Efficiency in Visible-Light-Induced NADH Regeneration by Intramolecular Electron Transfer. *ACS Appl. Mater. Interfaces* **2022**, *14* (34), 38895–38904. https://doi.org/10.1021/acsami.2c11174.

(32) Cobb, S. J.; Pornrungroj, C.; Andrei, V.; Badiani, V. M.; Su, L.; Manuel, R. R.; Pereira, I. A. C.; Reisner, E. A Photoelectrochemical-Thermoelectric Device for Semi-Artificial CO_2 Fixation Employing Full Solar Spectrum Utilization. *Device,* **2024**, *2* (11), 100505. https://doi.org/10.1016/j.device.2024.100505.

End of Supporting Information
